# Maternal Dietary Patterns during Pregnancy and Child Autism-Related Traits: Results from Two US Cohorts

**DOI:** 10.3390/nu14132729

**Published:** 2022-06-30

**Authors:** Rachel Vecchione, Siwen Wang, Juliette Rando, Jorge E. Chavarro, Lisa A. Croen, M. Daniele Fallin, Irva Hertz-Picciotto, Craig J. Newschaffer, Rebecca J. Schmidt, Kristen Lyall

**Affiliations:** 1 Department of Epidemiology and Biostatistics, Dornsife School of Public Health, Drexel University, Philadelphia, PA 19104, USA; rv422@drexel.edu; 2 Department of Nutrition, Harvard T.H. Chan School of Public Health, Harvard University, Boston, MA 02215, USA; siwenwang@hsph.harvard.edu (S.W.); jchavarr@hsph.harvard.edu (J.E.C.); 3 AJ Drexel Autism Institute, Drexel University, Philadelphia, PA 19104, USA; jr3639@drexel.edu; 4 Department of Epidemiology, Harvard T.H. Chan School of Public Health, Harvard University, Boston, MA 02215, USA; 5 Department of Medicine, Channing Division of Network Medicine, Brigham and Women’s Hospital and Harvard Medical School, Boston, MA 02215, USA; 6 Division of Research, Kaiser Permanente Norther California, Oakland, CA 94612, USA; lisa.a.croen@kp.org; 7 Department of Mental Health, Johns Hopkins, Baltimore, MD 21205, USA; dfallin@jhu.edu; 8 Department of Public Health Sciences, University of California Davis, Davis, CA 95616, USA; iher@ucdavis.edu (I.H.-P.); rjschmidt@ucdavis.edu (R.J.S.); 9 College of Health and Human Development, Pennsylvania State University, State College, PA 16801, USA; newschaffer@psu.edu

**Keywords:** dietary patterns, autism spectrum disorders, social responsiveness scale, AHEI-2010, AHEI-P, EDIP, aMED, prudent, western

## Abstract

We examined the relationship between maternal intake of established dietary patterns and child autism-related outcomes in two prospective cohorts in the United States. Participants were drawn from the Early Autism Risk Longitudinal Investigation (EARLI, *n* = 154) and the Nurses’ Health Study II (NHSII, *n* = 727). Dietary information was collected via food frequency questionnaires (FFQs) and used to calculate the empirical dietary inflammatory pattern (EDIP), Alternative Healthy Eating Index (AHEI), Western and Prudent dietary patterns, and the alternative Mediterranean Diet (aMED) score. Primary analyses examined associations with continuous autism-related traits as measured by the Social Responsiveness Scale (SRS), and secondary analyses with autism spectrum disorder (ASD) diagnosis. We used crude and multivariable quantile regression fixed at the 50th percentile to examine associations between quartiles of dietary patterns and SRS scores, and logistic regression to examine associations with ASD diagnosis. There was suggestion of a positive association with the Western diet (Q4 vs. Q1, ß = 11.19, 95% CI: 3.30, 19.90) in EARLI, though the association was attenuated with adjustment for total energy intake, and no clear associations were observed with other dietary patterns and ASD diagnosis or SRS scores. Further work is needed to better understand the role of maternal dietary patterns in ASD and related outcomes.

## 1. Introduction

Autism spectrum disorder (ASD) is a neurodevelopmental condition defined by social communication deficits and restricted repetitive behaviors, which present along a heterogeneous spectrum [1]. ASD has a complex etiology, with both genetic and environmental contributors [2], and evidence for prenatal origins [3]. Research has linked maternal prenatal diet with ASD [4,5,6], with most of this work focused on a small number of individual nutrients and foods [4,5,6,7,8,9,10,11,12,13]. The majority of studies have suggested inverse associations with ASD, particularly for folic acid and vitamin D levels, though conflicting findings exist [5,6,14,15,16,17,18,19,20,21,22,23,24].

Focusing on individual foods and nutrients, however, may obscure the relation between diet and ASD. Dietary patterns have the advantage of capturing overall dietary behavior and interactions between individual foods and nutrients. Moreover, dietary patterns are often good predictors of key biological mechanisms implicated in ASD [25,26,27], including inflammation and methylation [26,28,29]. Nevertheless, only a handful of studies to date have considered prenatal diet more comprehensively in relationship to ASD. One cohort study in the US found an inverse association between maternal adherence to the Mediterranean diet and autistic behaviors [30]. Two case–control studies in China also suggested associations between maternal dietary patterns or foods and ASD diagnosis, one reporting higher odds of ASD with unbalanced maternal patterns of either mostly meat or mostly vegetable intake [14], and the other reporting an inverse association with maternal diets high in fruit and fish [31].

In order to address gaps in our understanding of the relationship between comprehensively considered maternal diet and ASD, we used data from two prospective cohorts, the Early Autism Risk Longitudinal Investigation (EARLI) and the Nurses’ Health Study II (NHSII). In addition to allowing for prospective investigation of maternal diet, these two populations were chosen in order to enable unique cross-cohort comparisons. EARLI is a relatively small, family-based multi-site US study that capitalized on the increased ASD recurrence rate in siblings of those with ASD in order to enable rigorous, detailed prospective data collections to learn more about ASD etiology. In contrast, the NHSII is a large, population-based US nationwide study that has prospectively followed registered nurses for decades to learn about a range of health outcomes. This multi-cohort comparative design thus allowed us to address novel comparisons in the study maternal diet and ASD, including potential differences due to background likelihood of ASD, which has been suggested to modify relationships with other risk factors for ASD [32].

## 2. Materials and Methods

### 2.1. Study Population 

The study population for this work draws from two prospective cohorts in the United States, EARLI and the NHSII. EARLI is an increased family likelihood of ASD cohort that enrolled women who already had a child with confirmed ASD and followed them through a subsequent pregnancy until their child reached 3 years of age [33]. Eligibility criteria included being less than 29 weeks pregnant, speaking English or Spanish, being 18 or older, and living within two hours of Drexel/Children’s Hospital of Philadelphia (CHOP); Johns Hopkins University; University of California, Davis; or Northern California Kaiser Permanente. A total of 256 women were enrolled, and children were followed from 2009 to 2012. To be included in analyses, information on prenatal diet and child ASD-related outcomes was required. In addition, we did not include twins (*n* = 8 pairs) in order to avoid repeat diet information for the same pregnancy. Following these exclusions, 154 participants from EARLI were included in the present analyses (Figure S1).

The Nurses’ Health Study II (NHSII) is a large ongoing longitudinal study of 116,429 female registered nurses from the United States aged 25–42 years beginning in 1989 [34]. Biennial questionnaires are mailed to participants to inquire about lifestyle practices, reproductive events, and medical conditions. (All NHSII questionnaires are available online: https://nurseshealthstudy.org/participants/questionnaires; accessed on 9 June 2022). The average follow-up rate exceeds 90%. Participants were eligible for this current study if they had index births between 1991 and 2007, in order to allow for prospective reporting of diet and to include participants from the nested case–control study of ASD that collected the outcome measure used here. The details of this nested case–control study have been previously described [35]. Briefly, in 2005, all cohort participants were asked whether any of their children had autism, Asperger syndrome, or "other autism spectrum". In 2007, we enrolled 656 ASD cases and 2800 controls. Controls were randomly selected, and frequency matched at a ratio of 4:1 for years in which case mothers reported births (as, at the time, no information on case year of birth was available). After excluding women without eligible dietary or child information, 10,314 women completed a diet assessment during pregnancy or lactation. Of these, 727 women were part of the nested case–control study and returned an SRS form for their child (Figure S1).

Mothers/caregivers in EARLI gave written informed consent to study procedures, which were reviewed and approved by local site IRBs. In NHSII, return of questionnaires constitutes implied consent, and the study protocol of the parent NHSII was approved by the Institutional Review Boards of the Brigham and Women’s Hospital and the Harvard T.H. Chan School of Public Health.

### 2.2. Dietary Assessment

Dietary information in both studies was collected via comparable food frequency questionnaires (FFQs), both enabling calculation of dietary patterns. EARLI used a modified version of the National Cancer Institute’s Dietary History Questionnaire (DHQ). Modifications were made to assess diet during pregnancy and to capture additional information on toxicant exposures (e.g., added questions about organic intake and canned foods). Dietary information was collected at about the 20th and 36th week of pregnancy, covering diet during weeks 1–20 and 21–36 of gestation, respectively. Approximately 77.5% of participants had dietary information available at both time points, and prior work has indicated strong correlation between reported foods across these times [36]. In EARLI, the DHQ from the first timepoint in pregnancy was used in these analyses given the strong correlation across times and that most participants responded to this questionnaire.

In the NHSII, dietary intake was collected in 1991 and every 4 years thereafter, using a previously validated semiquantitative 131-item FFQ [37,38]. Participants were asked about the frequency of food consumption of a standard portion size of each food on average over the preceding year. An eligible dietary intake measure was defined as returning a valid FFQ one year prior to the birth year of the index child (termed as "pregnancy group" hereafter), or 1–2 years after birth of the index child and reported breastfeeding (termed as "lactation group" hereafter). In NHSII, all FFQs returned by participants during 1991–2007 were used to compute dietary patterns. In both studies, total energy intake was calculated by summing energy from all foods. We confirmed that individuals in each study did not have implausible energy intake values.

### 2.3. Dietary Pattern Indices

Dietary patterns, including the empirical dietary inflammatory pattern (EDIP), the Alternative healthy Eating Index (AHEI-2010), the Alternate Healthy Eating Index Modified for Pregnancy (AHEI-P), the Western and Prudent dietary patterns, and the Alternative Mediterranean Diet Score (aMED), were derived in each cohort according to standard approaches [39,40,41,42,43]. Because alcohol intake is associated with maternal and fetal outcomes, alcohol bearing groups were not included in dietary patterns here [44]. 

The EDIP was developed using reduced rank regression to summarize the overall inflammatory potential of diet based on intake of food groups shown to predict plasma concentration of three inflammatory markers: interleukin-6 (IL-6), C-reactive protein (CRP), and tumor necrosis factor α receptor 2 (TNFRα2) [28,39]. The Alternative Healthy Eating Index (AHEI) is a dietary pattern that characterizes adherence to dietary guidelines [45], and was updated in 2010 (AHEI-2010) to include new dietary factors (such as whole grains, sugar sweetened beverages and fruit juice, processed meat and sodium) [43]. In addition, we calculated the modified for pregnancy version of the AHEI (AHEI-P), which excludes foods not recommended during pregnancy such as alcohol and includes incorporation of folate, iron, and calcium [40]. The Western and Prudent dietary patterns were derived using principal component analysis from dietary information collected by FFQs based on cumulative average consumption of food/beverage groups, following prior work in the NHS cohort [40,41]. The Western pattern was characterized by high consumption of red and processed meat, butter, high-fat dairy products, eggs, and refined grains, while the Prudent patten was characterized by high consumption of fruits, vegetables, legumes, whole grains, fish and other seafood [41]. The aMED was developed to characterize adherence to a Mediterranean diet, tailored to a U.S. population [42]. For all dietary patterns, a higher score represents higher adherence to the pattern.

### 2.4. Outcome Assessment 

In both studies, ASD-related traits were measured using the Social Responsiveness Scale (SRS), a widely used and validated measure of ASD-related traits [46,47]. The SRS is a 65-item informant report measure (here, completed by mothers) that yields a single quantitative score, with higher scores indicating greater ASD-related traits and poorer social reciprocity [46,47]. Our analyses used total raw SRS scores as the primary outcome measure. Sex-normed T-scores, typically used to inform clinical settings, were also used in secondary analyses [46]. For forms with <10% missing, per publisher guidelines, item population median values were substituted for missing values. In EARLI, all outcome information, including SRS scores and ASD diagnostic evaluations, was collected at age 3, and EARLI, therefore, administered the preschool version of the SRS. In NHSII, child age ranged from 4–18, and the school-aged SRS was used as part of a nested case–control study [35]. Scoring procedures and value ranges were the same across these versions of the SRS; only minor wording changes differed for age-appropriate examples. Prior work has supported the stability of SRS scores across age [48].

Information on autism diagnosis was also available in both studies. In EARLI, autism diagnosis was defined according to clinical assessment and scores on the Autism Diagnostic Observation Schedule (ADOS) [32,49,50]. In NHSII, ASD diagnosis was defined according to maternal report of ASD on biennial follow-up questionnaires, as defined in prior work [35]. Maternal report of ASD in this medically trained population has been validated in a subset of participants administered the Autism Diagnostic Interview-Revised (ADI-R), with results suggesting a high degree of accuracy [35,51].

### 2.5. Statistical Analysis 

Data from both cohorts was analyzed in parallel using a common analytical plan. We used descriptive statistics to examine dietary intake in both cohorts and to determine associations with covariates, overall and by quartiles, of dietary patterns. In order to assess associations between maternal dietary patterns and child SRS scores, we used crude and multivariable adjusted quantile regression. Quantile regression allows for assessment of the relationship between exposure and outcome across quantiles of the outcome and also has the benefit over linear regression of being more robust to non-normally distributed outcomes [52]. Our primary results are shown for associations fixed at the 50th percentile. Dietary patterns were parameterized in quartiles with the lowest quartile as the referent. Covariates considered as potential confounders in adjusted models, based on *a priori* associations with outcomes and potential or known associations with maternal diet, included maternal age (continuous), child sex (male, female), maternal pre-pregnancy BMI (continuous), household income (EARLI: 0–50 k, 50–100 k, 100 k+; NHSII:0–40 k, 40–100 k, 100 k+), physical activity (METs/week), child year of birth (in NHSII given the larger distribution), maternal race (white, non-white), maternal prenatal smoking (active smoker, non-active smoker), prenatal vitamin use (yes, no), parity (continuous), and total energy intake (continuous). Final fully adjusted models did not include those variables with minimal impact on estimates (<10% change: smoking, race, physical activity, year of birth, and parity).

We also ran a series of secondary and sensitivity analyses. To compare to associations with ASD diagnosis, logistic regression was used to examine the association of each dietary pattern with ASD diagnosis. Primary analyses were also conducted using SRS T-scores rather than raw scores, in order to facilitate clinical interpretation. To examine whether the association between maternal diet and offspring SRS score differed by severity of autism traits, we fit multivariable quantile regression models fixed at other quantiles (10th, 25th, 75th, 90th). Modification by child’s sex was examined for primary associations (dietary patterns and SRS scores) using stratified models. In addition, because the NHSII did not explicitly measure dietary intake during pregnancy, but rather captured intake overlapping across pregnancy and lactation periods, we stratified analyses for this cohort by the derived timing of dietary assessment. Associations were examined separately in these subgroups (pregnancy and lactation). All statistical analyses were conducted using SAS 9.4.

## 3. Results

Sociodemographic characteristics of the study participants overall and by cohort are shown in Table 1. EARLI had higher racial and ethnic diversity than NHSII, though the majority of participants in both cohorts were non-Hispanic White. Fewer participants were in the lowest income category in NHSII than in EARLI. As expected, because of the familial nature of the cohort, participants in EARLI had a higher mean SRS raw score (35.8 vs. 28.2) than participants in NHSII. Distributions of SRS scores in both cohorts are shown in Figure S2 and are comparable to prior studies. Characteristics of the two subgroups in NHSII were similar (Table S1). Characteristics were also similar across quartiles of Western dietary pattern (as well as the EDIP, aMED, and Prudent), though in individuals in the highest quartile of the AHEI-2010 compared to those in the lowest quartile, maternal age was slightly higher in both cohorts, and BMI lower in EARLI (Table S2). The distribution of dietary pattern scores was highly comparable across cohorts (Table S3). In both cohorts, total energy intake was positively associated with quartiles of dietary pattern, and this relationship was strongest for the Western dietary pattern (Table S4).

In analyses examining the associations between maternal dietary patterns during pregnancy and child SRS scores in EARLI, overall, strong associations were not observed (Table 2). Estimates for top quartiles for most dietary patterns, which broadly represent an overall healthier diet for the Prudent, AHEI, and aMED, and overall less healthy diet or more pro-inflammatory diet for the Western and EDIP, respectively, were below the null for all patterns except the Western diet. Statistically significant increases in SRS scores were observed across all quartiles of intake relative to the lowest quartile in the adjusted model not including total energy intake, though estimates were similar across quartiles (no evidence for trend) and additional adjustment for total energy intake attenuated estimates (only Q2 vs. Q1 remained statistically significant (ß = 11.71, 95% CI: 2.16, 22.71). Adjusted estimates for the top quartiles (Q4) of both the Prudent and the aMED dietary patterns were suggestive of an inverse relationship (ß = −14.17, 95% CI: −22.76, 2.11; ß = −12.32, 95% CI: −23.33, 4.05).

In NHSII, estimates across quartiles of the EDIP, AHEI, Prudent, and Mediterranean dietary patterns were generally null (Table 3). There were slight increases in SRS scores for Q2 and Q4 of the Western dietary pattern in the adjusted model not including total energy intake, but these increases did not remain statistically significant with adjustment for total energy intake, and, as in EARLI, no trend was suggested. In both EARLI and NHSII, adjustment for additional covariates, including physical activity, did not materially alter findings (Tables S5 and S6).

In analyses examining associations with ASD diagnosis, results were generally consistent with those of SRS scores, suggesting no strong associations with these maternal dietary patterns in both cohorts (Table S7, EARLI, and Table 4, NHSII). In EARLI, adjusted ORs were above the null for intake above the median (quartiles collapsed due to small *n* values) for both the AHEI-2010 and the aMED, but with wide CIs. In NHSII, there was a non-significant increased odds of ASD with higher EDIP (Q4 vs. Q1 AOR = 1.41, 95% CI 0.76, 2.62), and mainly null estimates for other dietary patterns.

Across additional secondary analyses, results were broadly consistent with primary findings. Findings were consistent with primary analyses when using T-scores (Table S8). In both EARLI and NHSII, while there were some suggestions of higher estimates with increasing quantile of SRS across percentiles in quantile regression (Tables S9 and S10, Figures S3 and S4), confidence intervals were increasingly wide in these analyses. We did not observe evidence for effect modification by child’s sex (Table S11), and precision was low in these stratified models, especially in EARLI. Estimates were consistently further from the null in males than in females in both cohorts, though confidence intervals were wide. Examining associations in NHSII timing of diet subgroups, estimates were somewhat stronger in the pregnancy subgroup of the NHSII than in the lactation subgroup (Table S12), with increases in SRS scores for Q2 and Q4 of the Western dietary pattern in particular, but confidence intervals crossed the null.

## 4. Discussion

In this study drawing from two prospective longitudinal cohorts of more than 800 pregnant women in the United States, we did not find strong evidence that maternal dietary patterns were associated with ASD-related traits or diagnosis. This is the first study to examine relationships between a series of established dietary patterns in and surrounding pregnancy and child ASD-related outcomes. Our cross-cohort comparative design also enabled consideration of the role of background familial likelihood of ASD in these relationships. While strong associations were not identified in either population, there were some suggestions of associations in the familial cohort that were not evident in the general population cohort. Given the known importance of prenatal and early life nutrition to child health and development, continued work should address how these dietary patterns and other dietary factors may influence ASD and related outcomes in other ways than assessed here.

Few prior studies have addressed the role of prenatal dietary patterns and ASD-related outcomes. A 2018 case–control study in China, including 708 participants, 354 of whom had a confirmed autism diagnosis, examined maternal dietary patterns during and prior to pregnancy in relationship to ASD case status [14]. This study did not utilize established dietary patterns as addressed here, but found that, compared to a diet balanced in meat and vegetable intake, “unbalanced” diets high in either meat or vegetables were associated with increased odds of ASD. Both the EDIP and Western dietary patterns have meat as a key component, and thus our suggestions of some signals with these patterns could be consistent with their findings [14]. In another study including 325 mother–child pairs enrolled in the US-based Newborn Epigenetics STudy (NEST), high maternal periconceptional adherence to a Mediterranean diet was associated with lower odds of autistic behavior as measured by composite scores from the Infant-Toddler Social and Emotional Assessment [30]. While we did not observe significant associations with the Mediterranean diet here, estimates for the top quartile of the aMED, as well as the Prudent diet (which shares some similarity to a Mediterranean-style diet) in EARLI (but not NHSII) were suggestive of an inverse relationship. These findings are of additional interest given evidence that low adherence to a Mediterranean dietary pattern has been associated with increased methylation in a sex-specific manner [29], and given links between methylation, folic acid, and ASD [53,54]. The potentially stronger effects in EARLI as compared to NHSII for these patterns could suggest possible interactions or differences based on background risk. Finally, a small case–control study in China including 295 participants, 108 of whom had ASD, found that diets high in fruit and those high in fish were associated with lower odds of ASD [31]. In comparison, we saw null associations between the AHEI, AHEIP dietary patterns and ASD and ASD-related traits, all of which include fruit and fish as essential components and may characterize a diet high in fruit or fish. However, prior work in the EARLI study has identified an association between fish intake and SRS scores that differed by timing and type of fish [9]. Thus, both similarities and discrepancies across these few studies exist. Differences may be related to assessment and characterization of dietary intake, as well as to time periods for which diet represents.

In our study, we observed some suggestions of a link between unhealthy diet according to the Western diet and increases in ASD-related outcomes. This relationship was stronger in EARLI, and when not adjusting for total energy intake. The Western dietary pattern had the strongest correlation with total energy intake, and variability in total energy intake was also highest in the highest quartile of dietary patterns, which may have contributed to the less precise estimates in these energy-adjusted models. It is also possible that total consumption may be a causal intermediate of the relationship between diet and neurodevelopment, or may have joint effects with other covariates, such as BMI or income. We did not have sufficient sample size in EARLI, where Western pattern associations were primarily seen, to adequately address potential interactions. Prior work has suggested a positive relationship between maternal obesity and gestational weight gain and ASD, and thus weight, increased caloric intake, and unhealthy intake may together influence these outcomes. There was also a non-significant elevation in odds of ASD diagnosis with higher EDIP in the NHSII. Both the EDIP and the Western dietary patterns include pro-inflammatory food groups such as processed and red meat, and high adherence to both dietary patterns is consistent with a pro-inflammatory diet [39,41]. Pro-inflammatory diets have been shown to be associated with a higher risk of adverse pregnancy outcomes [55], and accumulating evidence suggests prenatal immune disruption in ASD, including microglial (immune cells in the brain involved in neural circuitry development) dysfunction, inflammation in postmortem brain studies and studies of prenatal cytokine levels, and increased risk of ASD following maternal immune-mediated conditions during pregnancy [56,57,58]. Further work addressing potential links with pro-inflammatory dietary factors, therefore, seems warranted.

There are several potential explanations for the lack of strong associations in our work. One may be due to these being relatively healthy cohorts. There was a relatively high degree of supplement use in these cohorts. It is possible that in less healthy, more nutrient-deficient populations, the association between dietary intake and ASD-related outcomes may be stronger. It is also possible that associations with prenatal diet are tied to more specific phenotypic aspects or subgroups than examined here. Some prior work examining maternal dietary factors, for example, has suggested stronger associations between maternal polyunsaturated fatty acid levels and ASD with co-occurring intellectual disability [10,11]. Diet may also interact with other environmental exposures [59], and thus null independent effects do not necessarily imply lack of overall importance, particularly considering evidence for joint effects in key pathways [29].

Our study has several strengths, including the ability to examine multiple established dietary patterns in association with both ASD-related traits and diagnosis in both an increased familial likelihood (EARLI) and general population cohort (NHSII). Additional strengths include clinical assessments in EARLI, the use of established FFQs [37,38], and our ability to adjust for a variety of variables. However, several limitations of our study should also be noted and considered as opportunities for future work. Our sample size, while in line with or larger than prior studies, was relatively small, particularly in EARLI. This may have limited our ability to detect associations and what may have been more modest differences by background familial likelihood. Furthermore, while we hypothesize that the modest differences in cohort results may be due to this differing background likelihood, and we have ensured similarity of exposure and outcome measurements, we cannot rule out other study design or population differences playing into such cross-cohort comparisons. Dietary intake information in NHSII was not measured during pregnancy for all participants. Results were broadly similar in analysis stratified by pregnancy and lactation, but were somewhat stronger for the pregnancy group. The timing of EARLI dietary data represented early pregnancy (first 20 weeks), while NHSII spanned a larger period of time also outside of pregnancy, and thus results may have been biased towards the null if the critical window of these dietary factors was more specifically tied to late pregnancy (a period of rapid brain growth and uptake of key nutrients such as PUFAs). Prior research has suggested pre- and during-pregnancy diet was fairly stable in the NHSII [60,61]; nonetheless, timing may be an important future consideration. In addition, while prior work has validated these and other FFQs and our goal was to summarize associations with patterns of usual intake, reported diet is known to be measured with error, and we did not have biomarker-based comparisons with which to validate intake. Future work examining multiple nutrients and complex dose–response relationships may benefit from the addition of biomarker and metabolomics data. Finally, we cannot rule out potential residual confounding by aspects such as maternal phenotypic factors that may influence dietary habits (as suggested in some work [62]) and that also relate to child ASD-related traits; however, supplemental analyses adjusting for maternal SRS scores where available did not change conclusions.

## 5. Conclusions

Dietary patterns represent useful tools for understanding and communicating how usual intake of foods relates to outcomes. Patterns more closely assess diet as it is experienced, and also help capture combined effects of dietary factors. Though we did not observe strong associations between maternal dietary patterns and ASD-related outcomes in the two cohorts studied here, future work should consider following up on suggestive findings. This includes addressing potential associations with more pro-inflammatory or unhealthy diets and examining the role of diet in potential critical windows of neurodevelopment. In addition, extensions of this work addressing how dietary patterns may modify associations with other risk factors that share the same pathways are needed.

## Figures and Tables

**Table 1:** Basic characteristics of the NHSII (*n* = 727) and EARLI (*n* = 154) study populations.

	EARLI	NHSII ^1^
	*n* (%)	
Child sex		
Male	83 (53.9)	423 (58.2)
Female	71 (46.1)	304 (41.8)
Maternal ethnicity		
Hispanic/Latino	30 (19.5)	14 (1.9)
Not Hispanic/Latino	124 (80.5)	713 (98.1)
Maternal race		
White	104 (67.5)	708 (97.4)
Black/African American	9 (5.8)	1 (0.1)
Native American or Native Alaskan	2 (1.3)	
Asian & Pacific Islander	20 (13.0)	9 (1.2)
Multiple/Other Race	14 (9.1)	7 (1.0)
Other/unknown	5 (3.3)	2 (0.3)
Household income		
$0–50,000/$0–40,000	36 (23.4)	27 (3.7)
$50,001–100,000/$40,001–100,000	58 (37.7)	316 (43.5)
$100,001+	60 (39.0)	249 (34.3)
Missing	0 (0)	135 (18.6)
Prenatal smoking		
Active	5 (3.3)	51 (7.0)
Not active	122 (79.2)	676 (93.0)
Missing	27 (17.5)	0 (0)
Birthweight		
Low	9 (5.8)	7 (1.0)
Normal	143 (92.9)	436 (60.0)
Missing	2 (1.3)	284 (39.1)
Ever breastfeed		
Yes	98 (63.6)	691 (95.1)
No	37 (24.0)	35 (4.8)
Missing	19 (12.3)	1 (0.1)
Prenatal vitamin use		
Yes	145 (94.2)	532 (73.2)
No	8 (5.2)	194 (26.7)
Missing	1 (0.7)	1 (0.1)
Prenatal vitamin use (first month)		
Yes	88 (57.1)	-
No	65(42.2)	-
Missing	1 (0.7)	-
ASD diagnosis		
Yes	32 (20.8)	106 (14.6)
No	120 (77.9)	621 (85.4)
Missing	2 (1.3)	0 (0)
	**Mean (std)**	
Maternal age, years	33.9 (4.6)	34.2 (4.2)
Birthweight (lb)	7.6 (1.2)	-
Parity	1.8 (0.9)	1.3 (1.2)
Pre-Pregnancy BMI, kg/m^2^	28.0 (7.1)	23.4 (4.2)
Physical activity, METs/week	366.8 (617.8)	20.3 (26.1)
Total caloric intake, kcal	1828.9 (801.4)	1946.2 (549.4)
Total SRS raw score	35.8 (26.2)	28.2 (34.0)

Abbreviations: EARLI: Early Autism Risk Longitudinal Investigation, NHSII: Nurses’ Health Study II, SRS: Social Responsiveness Scale, ASD: Autism Spectrum Disorder, BMI: Body Mass Index, METs: Metabolic Equivalents. ^1^ Characteristics shown for the full NHSII group (diet during pregnancy or lactation). Characteristics by NHSII subgroups are provided in Table S1.

**Table 2:** Association between maternal dietary patterns during pregnancy and child SRS raw scores in EARLI (*n* = 131).

	*n*	Crude (ß, 95% CI)	Adjusted (ß, 95% CI)	Fully Adjusted (ß, 95% CI)
EDIP				
Q1	31	0 (reference)	0 (reference)	0 (reference)
Q2	35	−4.00 (−17.53, 6.02)	−8.42 (−15.22, 3.01)	−8.29 (−14.49, 3.02)
Q3	29	−10.00 (−16.99, 0.66)	−8.72 (−16.65, 4.04)	−7.72 (−15.58, 3.80)
Q4	36	3.00 (−8.61, 11.08)	−4.60 (−9.47, 5.37)	−6.70 (−10.71, 6.55)
AHEI-2010				
Q1	35	0 (reference)	0 (reference)	0 (reference)
Q2	40	−11.00 (−22.55, 2.10)	−6.14 (−13.45, 4.44)	−6.30 (−14.38, 3.91)
Q3	24	−7.00 (−20.47, 5.47)	−0.08 (−8.68, 5.41)	−5.86 (−11.45, 5.10)
Q4	32	−8.00 (−19.18, 3.55)	−2.97 (−18.22, 9.52)	−6.03 (−23.11, 11.96)
AHEI-P				
Q1	34	0 (reference)	0 (reference)	0 (reference)
Q2	33	1.00 (−10.29, 11.10)	7.98 (−7.47, 14.08)	3.70 (−10.91, 14.04)
Q3	34	3.00 (−22.32, 13.08)	7.80 (−5.52, 12.92)	0.02 (−16.34, 17.43)
Q4	30	−1.00 (−14.70, 8.85)	2.12 (−13.78, 7.12)	−7.58 (−25.64, 9.14)
Western ^†^				
Q1	30	0 (reference)	0 (reference)	0 (reference)
Q2	32	11.00 (2.21, 21.89)	12.87 (4.46, 22.59)	11.71 (2.16, 22.71)
Q3	33	3.00 (−6.69, 13.42)	7.55 (0.62, 15.81)	5.29 (−6.85, 16.77)
Q4	36	14.00 (−3.01, 24.00)	11.19 (3.30, 19.90)	8.01 (−19.90, 22.14)
Prudent ^†^				
Q1	33	0 (reference)	0 (reference)	0 (reference)
Q2	35	−5.00 (−20.16, 6.31)	−6.45 (−10.78, 8.51)	−7.69 (−13.31, 7.30)
Q3	31	0.00 (−9.91, 10.82)	−1.59 (−11.46, 15.39)	−6.04 (−12.14, 14.62)
Q4	32	−7.00 (−19.95, 8.90)	−9.54 (−18.97, 5.62)	−14.17 (−22.76, 2.11)
aMED				
Q1	20	0 (reference)	0 (reference)	0 (reference)
Q2	55	−7.00 (−19.58, −0.21)	−8.29 (−13.11, 4.74)	−9.02 (−12.88, 5.85)
Q3	27	−1.00 (−12.41, 5.71)	1.53 (−3.40, 12.08)	0.24 (−8.50, 9.99)
Q4	29	−8.00 (−21.62, 4.81)	−8.31 (−14.03, 5.65)	−12.32 (−23.33, 4.05)

Abbreviations: EDIP: Empirical Dietary Inflammatory Pattern, AHEI: Alternative Healthy Eating Index, AHEI-P: Alternative Healthy Eating Index Modified for Pregnancy, aMED: Alternative Mediterranean Diet Score. Q stands for quartile, e.g., Q1 = quartile 1 (lowest quartile of intake for a given dietary pattern, Q4 = highest quartile for a given pattern). Modeled using quantile regression fixed at 50th percentile. Adjusted: Adjusted for maternal age (continuous), child sex (male, female), maternal pre-pregnancy BMI (continuous), household income (0–50 k, 50–100 k, 100 k+), prenatal vitamin use in first month (yes, no). Fully Adjusted: Adjusted Model, additionally adjusted for total energy intake (continuous). ^†^ Mutually adjusted for each other.

**Table 3:** Association between maternal dietary patterns and child SRS raw scores in the NHSII (*n* = 727).

	*n*	Crude (ß, 95% CI)	Adjusted (ß, 95% CI)	Fully Adjusted (ß, 95% CI)
EDIP				
Q1	167	0 (reference)	0 (reference)	0 (reference)
Q2	200	−3.00 (−10.36, 8.36)	−3.92 (−6.78, −0.54)	−3.94 (−6.71, −0.58)
Q3	175	−3.00 (−9.57, 9.57)	−1.82 (−5.13, 0.76)	−1.92 (−4.44, 1.17)
Q4	185	0.00 (−9.70, 12.70)	1.04 (−2.79, 3.76)	0.96 (−2.74, 3.17)
AHEI-2010				
Q1	199	0 (reference)	0 (reference)	0 (reference)
Q2	178	3.00 (−8.52, 11.02)	0.33 (−1.40, 3.70)	0.36 (−2.78, 4.22)
Q3	175	3.00 (−8.97, 6.97)	0.19 (−3.12, 3.16)	0.13 (−3.61, 3.46)
Q4	175	5.00 (−9.97, 10.97)	2.21 (−1.35, 5.89)	2.62 (−2.03, 6.93)
AHEI-P				
Q1	193	0 (reference)	0 (reference)	0 (reference)
Q2	169	−2.00 (−6.82, 9.82)	−0.49 (−4.29, 1.99)	−1.31 (−5.51, 1.72)
Q3	174	−1.00 (−10.89, 9.89)	−0.80 (−4.30, 1.31)	−1.61 (−4.53, 1.06)
Q4	191	1.00 (−7.12, 13.12)	2.69 (−2.12, 6.49)	1.51 (−3.32, 4.44)
Western ^†^				
Q1	168	0 (reference)	0 (reference)	0 (reference)
Q2	178	5.00 (−9.18, 16.18)	2.79 (0.19, −7.71)	2.71 (−0.31, 7.73)
Q3	188	3.00 (−8.19, 12.69)	2.50 (−1.05, 6.94)	1.68 (−2.46, 6.78)
Q4	193	5.00 (−8.20, 14.41)	3.34 (0.39, 7.99)	1.83 (−2.35, 9.38)
Prudent ^†^				
Q1	197	0 (reference)	0 (reference)	0 (reference)
Q2	182	−2.00 (−11.00, 11.50)	−0.73 (−5.05, 2.04)	−1.09 (−4.81, 2.89)
Q3	170	0.00 (−8.70, 14.35)	1.15 (−2.49, 3.65)	0.23 (−4.04, 3.93)
Q4	178	3.00 (−7.39, 15.39)	3.88 (−1.14, 7.10)	2.73 (−1.92, 8.16)
aMED				
Q1	170	0 (reference)	0 (reference)	0 (reference)
Q2	248	2.00 (−6.86, 12.86)	0.61 (−0.74, 4.01)	0.27 (−1.76, 3.87)
Q3	146	2.00 (−5.70, 12.40)	0.30 (−2.47, 4.38)	−0.49 (−4.75, 4.51)
Q4	163	2.00 (−4.95, 11.45)	3.10 (−0.77, 6.04)	2.16 (−3.70, 5.98)

Abbreviations: EDIP: Empirical Dietary Inflammatory Pattern, AHEI: Alternative Healthy Eating Index, AHEI-P: Alternative Healthy Eating Index Modified for Pregnancy, aMED: Alternative Mediterranean Diet Score. Q stands for quartile, e.g., Q1 = quartile 1 (lowest quartile of intake for a given dietary pattern, Q4 = highest quartile for a given pattern). Models using quantile regression fixed at 50th percentile. Adjusted: Adjusted for maternal age (continuous), child sex (male, female), maternal pre-pregnancy BMI (continuous), household income (0–40 k, 40–100 k, 100 k+), prenatal vitamin use (yes, no). Fully Adjusted: Adjusted model, additionally adjusted for total energy intake (continuous). ^†^ Mutually adjusting for each other.

**Table 4:** Association between maternal dietary patterns and child autism spectrum disorder diagnosis in NHSII (*n* = 727).

	Cases/*n*	Crude (RR, 95% CI)	Adjusted (RR, 95% CI)	Fully Adjusted (RR, 95% CI)
EDIP				
Q1	23/167	1 (reference)	1 (reference)	1 (reference)
Q2	24/200	0.85 (0.46, 1.58)	0.95 (0.50, 1.79)	0.96 (0.51, 1.81)
Q3	25/175	1.04 (0.57, 1.92)	1.04 (0.55, 1.96)	1.04 (0.55, 1.96)
Q4	34/185	1.41 (0.79, 2.51)	1.55 (0.85, 2.84)	1.41 (0.76, 2.62)
AHEI-2010				
Q1	27/199	1 (reference)	1 (reference)	1 (reference)
Q2	27/178	1.14 (0.64, 2.03)	1.06 (0.58, 1.93)	1.09 (0.60, 2.00)
Q3	25/175	1.06 (0.59, 1.91)	1.00 (0.54, 1.83)	1.07 (0.58, 1.99)
Q4	27/175	1.16 (0.65, 2.07)	1.02 (0.56, 1.87)	1.12 (0.60, 2.07)
AHEI-P				
Q1	27/193	1 (reference)	1 (reference)	1 (reference)
Q2	16/169	0.64 (0.33, 1.24)	0.62 (0.32, 1.23)	0.56 (0.28, 1.12)
Q3	27/174	1.13 (0.63, 2.01)	0.99 (0.54, 1.82)	0.84 (0.44, 1.60)
Q4	36/191	1.43 (0.83, 2.46)	1.30 (0.73, 2.33)	0.98 (0.50, 1.96)
Western ^†^				
Q1	26/168	1 (reference)	1 (reference)	1 (reference)
Q2	23/178	0.83 (0.45, 1.53)	0.78 (0.41, 1.47)	0.61 (0.32, 1.19)
Q3	27/188	0.91 (0.50, 1.64)	0.90 (0.48, 1.66)	0.55 (0.27, 1.14)
Q4	30/193	1.01 (0.57, 1.81)	0.91 (0.49, 1.69)	0.36 (0.14, 0.91)
Prudent ^†^				
Q1	32/197	1 (reference)	1 (reference)	1 (reference)
Q2	16/182	0.50 (0.26, 0.94)	0.48 (0.25, 0.94)	0.38 (0.19, 0.76)
Q3	25/170	0.89 (0.50, 1.57)	0.82 (0.45, 1.49)	0.57 (0.29, 1.10)
Q4	33/178	1.17 (0.68, 2.01)	1.09 (0.61, 1.93)	0.57 (0.27, 1.21)
aMED				
Q1	23/170	1 (reference)	1 (reference)	1 (reference)
Q2	30/248	0.88 (0.49, 1.57)	0.87 (0.47, 1.60)	0.79 (0.43, 1.47)
Q3	22/146	1.13 (0.60, 2.13)	0.95 (0.49, 1.84)	0.79 (0.40, 1.58)
Q4	31/163	1.50 (0.83, 2.70)	1.45 (0.78, 2.69)	1.19 (0.62, 2.31)

Abbreviations: EDIP: Empirical Dietary Inflammatory Pattern, AHEI: Alternative Healthy Eating Index, AHEI-P: Alternative Healthy Eating Index Modified for Pregnancy, aMED: Alternative Mediterranean Diet Score. Models using logistic regression. Adjusted: Adjusted for maternal age (continuous), child sex (male, female), maternal pre-pregnancy BMI (continuous), household income (0–40 k, 40 k–100 k, 100 k+), prenatal vitamin use (yes, no). Fully Adjusted: Adjusted model, additionally adjusted for total energy intake (continuous). ^†^ Mutually adjusting for each other.

## Data Availability

Data are available upon reasonable request and approval of EARLI study investigators. In addition, EARLI participates in the National Database for Autism Research (NDAR). Further information including the procedures to obtain and access data from the Nurses’ Health Studies is described at https://www.nurseshealthstudy.org/researchers (accessed on 9 June 2022; contact email: nhsaccess@channing.harvard.edu).

## Supplementary Materials

The following supporting information can be downloaded at: https://www.mdpi.com/article/10.3390/nu14132729/s1, Figure S1: Study Participant Flowchart across the EARLI and NHSII cohorts; Figure S2: Distribution of SRS Raw Scores in EARLI and NHSII; Table S1: Basic characteristics of NHSII subgroup pregnancy (*n* = 347) and lactation (*n* = 380); Table S2: Basic characteristics of the study population by high and low quartiles of AHEI-2010 across the two cohorts; Table S3: Distribution of dietary pattern scores in quartiles across the two cohorts; Table S4: Distribution of total energy intake (mean, SD) by dietary patterns in EARLI and NHSII; Table S5: Association between maternal dietary patterns during pregnancy and child SRS raw scores in EARLI, adjusted for additional potential confounders (*n* = 131); Table S6: Association between maternal dietary patterns and child SRS raw scores in NHSII, adjusted for additional potential confounders (*n* = 727); Table S7: Association between maternal dietary patterns during pregnancy and child autism spectrum disorders in EARLI (*n* = 146); Table S8: Association between maternal dietary patterns during pregnancy and child SRS T scores in EARLI (*n* = 131) and NHSII (*n* = 727); Table S9: Full quantile regression results for the association between maternal dietary patterns and child SRS scores in EARLI (*n* = 131); Table S10: Full quantile regression results for the association between maternal dietary patterns and child SRS scores in NHSII (*n* = 727); Figure S3. ß estimates from fully adjusted quantile regression plots demonstrating association between dietary pattern scores during pregnancy and child SRS raw scores in EARLI; Figure S4. ß estimates from fully adjusted quantile regression plots demonstrating association between dietary pattern scores during pregnancy and child SRS raw scores in NHSII; Table S11: Associations between maternal dietary patterns and child SRS scores in the NHSII and EARLI cohorts, stratified by child’s sex; Table S12: Association between maternal dietary patterns and child SRS raw scores in NHSII subgroups.
