# Supplementary material for: Maternal Dietary Patterns during Pregnancy and Child Autism-Related Traits: Results from Two US Cohorts
Source: Nutrients. 2022 Jun 30;14(13):2729. doi: 10.3390/nu14132729 (PMC9268965; doi:10.3390/nu14132729)
Supplement: Supplementary file 1 [file nutrients-14-02729-s001.zip › nutrients-1753228-supplementary.pdf]

Supplementary Materials

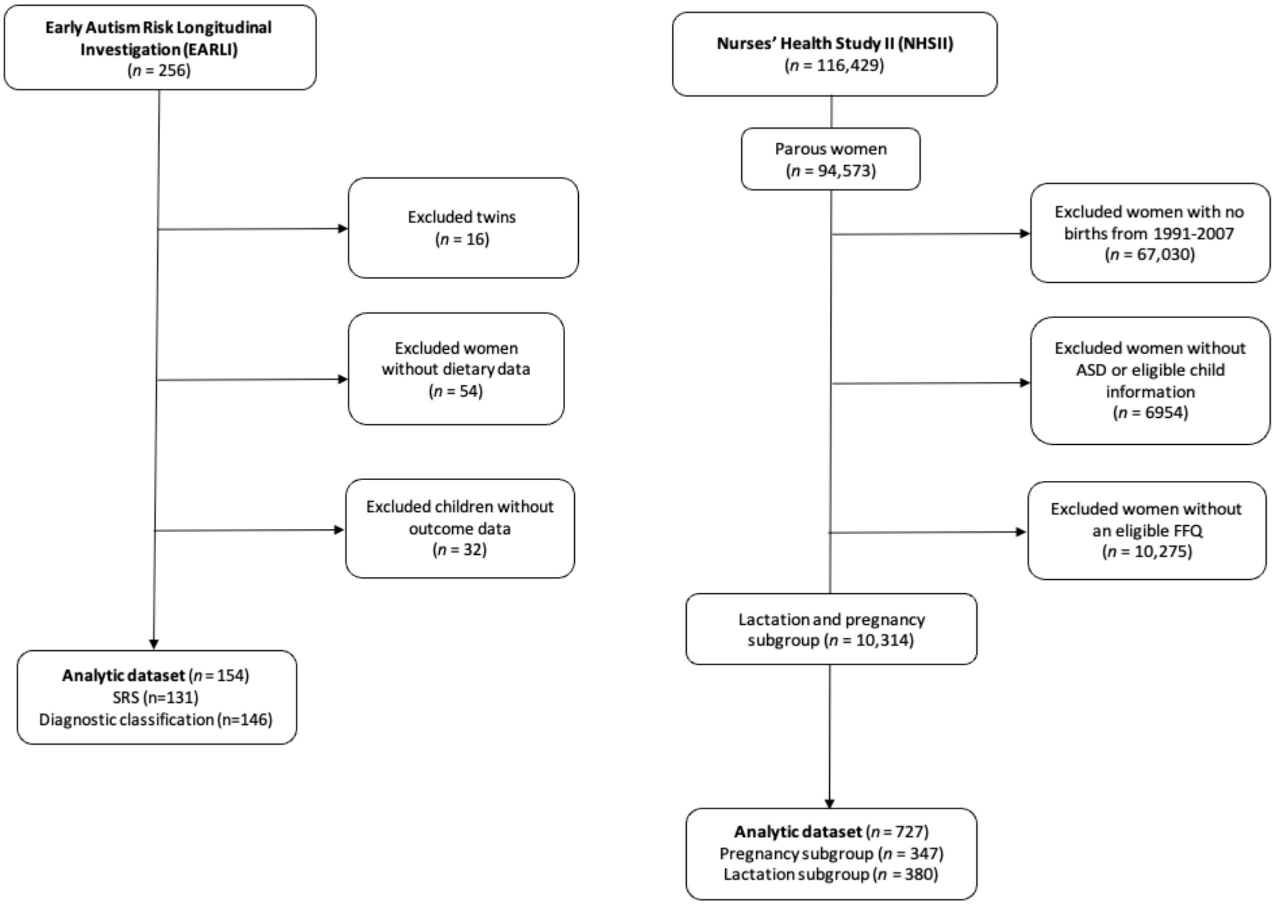

Figure S1. Study Participant Flowchart across the EARLI and NHSII cohorts. Figure S1 shows individual flow charts for each cohort included in the study analyses. A cross-cohort comparative approach was taken, so that data was not pooled across cohorts, but rather, results compared due to the differences in study populations. Slight differences in exclusions across cohorts are due to the differences in data collection procedures. In the Nurses Health Study II, births outside of 1991–2007 were not included in order to ensure prospective collection of information for use in these analyses. We also did not include women who were not eligible for the nested case-control study that collected the primary outcome measure used here, and thus women without ASD information available and those that had been excluded from our nested case control follow study due to missing year of birth, having an adopted child, or lack of confirmation of diagnoses, were excluded in order to ensure comparability and availability of prospectively collected information.

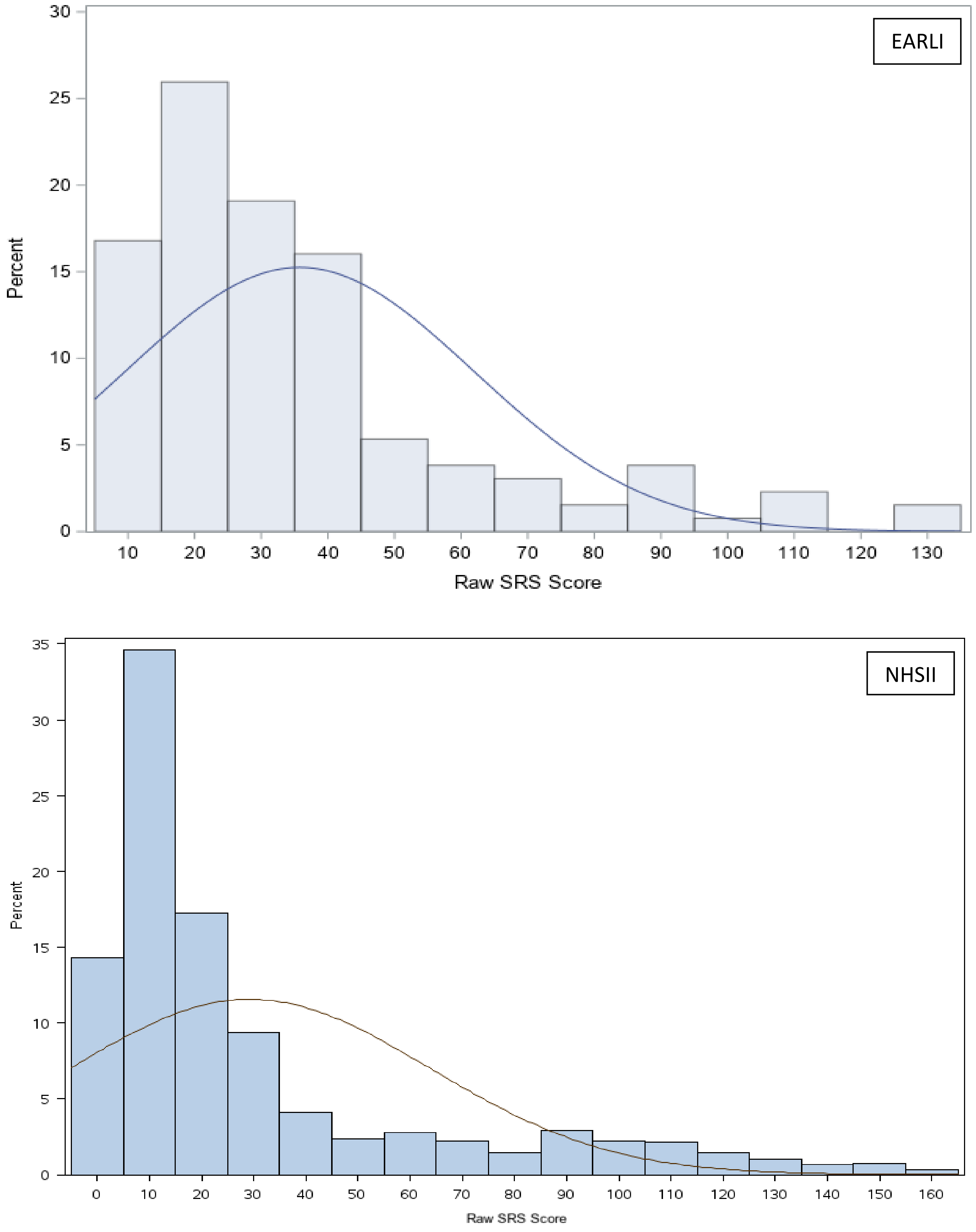

Figure S2: Distribution of Raw SRS scores in EARLI and NHSII

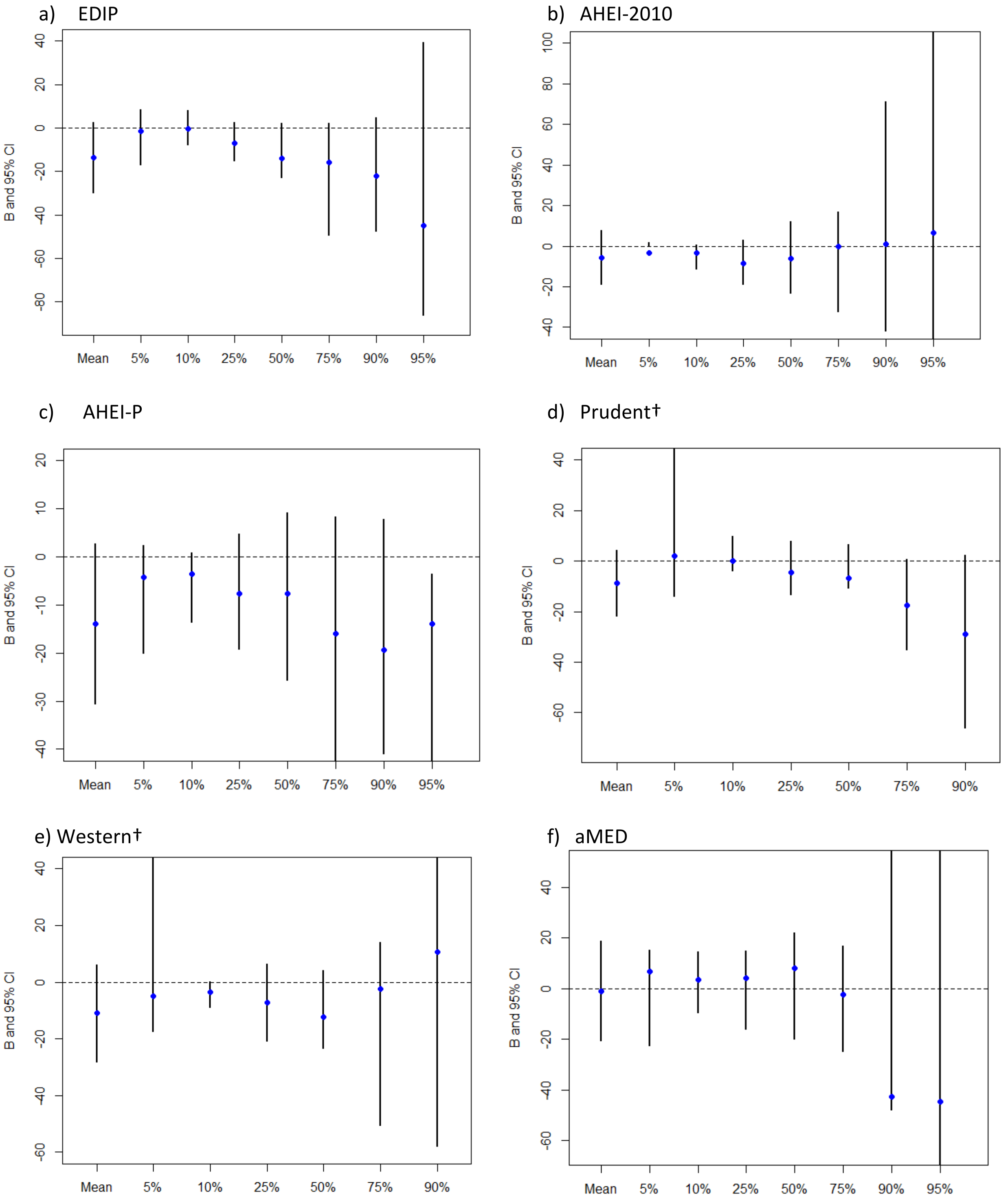

Figure S3. ß estimates [1] from fully adjusted quantile regression plots demonstrating association between dietary pattern scores during pregnancy and child SRS raw scores in EARLI.[2] Abbreviations: EDIP: Empirical Dietary Inflammatory Pattern, AHEI: Alternative Healthy Eating Index, AHEI-P: Alternative Healthy Eating Index Modified for Pregnancy, aMED: Alternative Mediterranean Diet Score
[1] Estimates for quartile 4 compared to quartile 1. [2] Blue dots indicate the ß from adjusted models including adjustment for maternal age (continuous), child sex (male, female), maternal pre-pregnancy BMI (continuous), household income (0–50k, 50k–100k, 100k+), prenatal vitamin use in first month (yes, no), and total energy intake (continuous). Black lines indicate 95% CIs. †Mutually adjusted for each other

a) EDIP

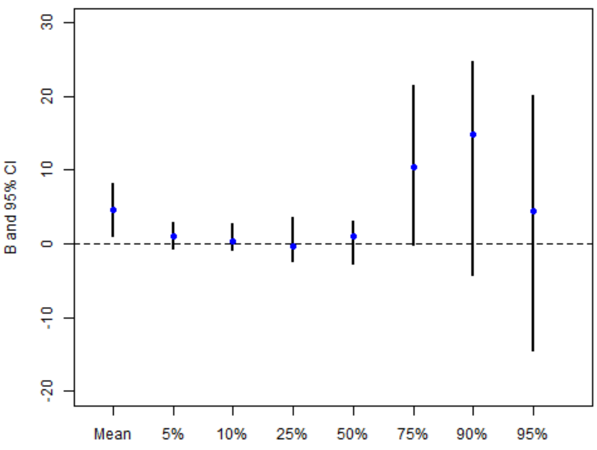

b) AHEI-2010

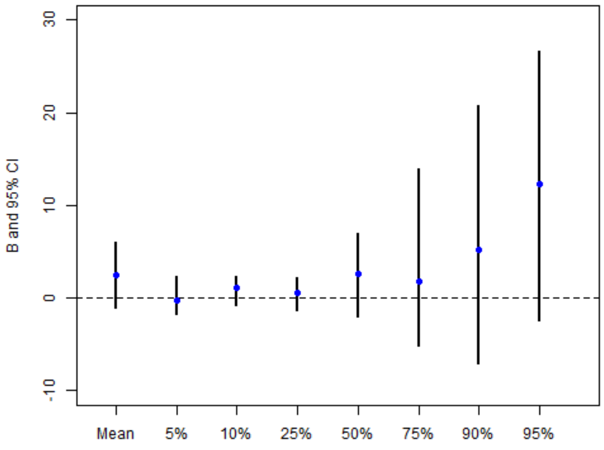

c) AHEI-P

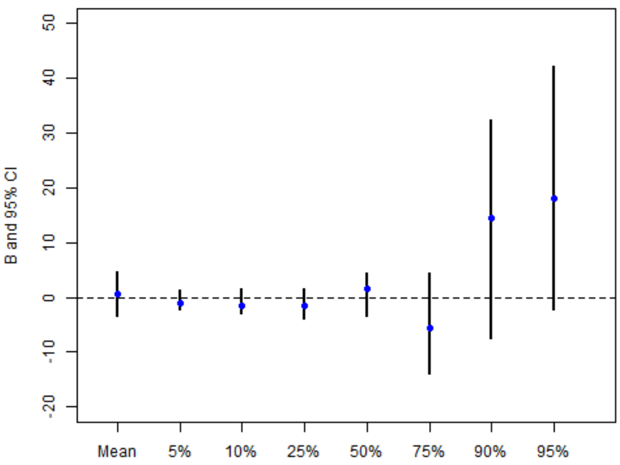

d) Prudent†

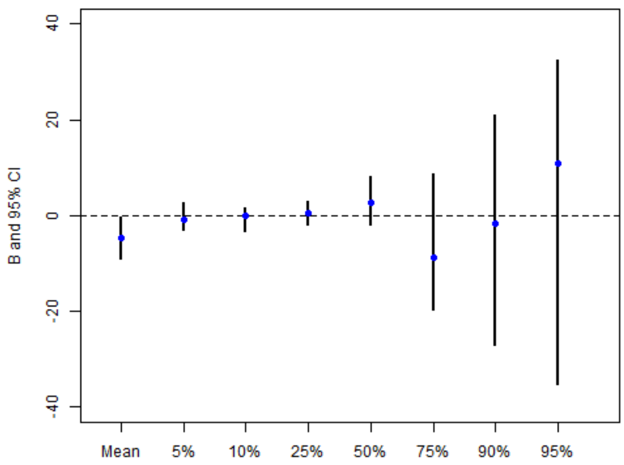

e) Western†

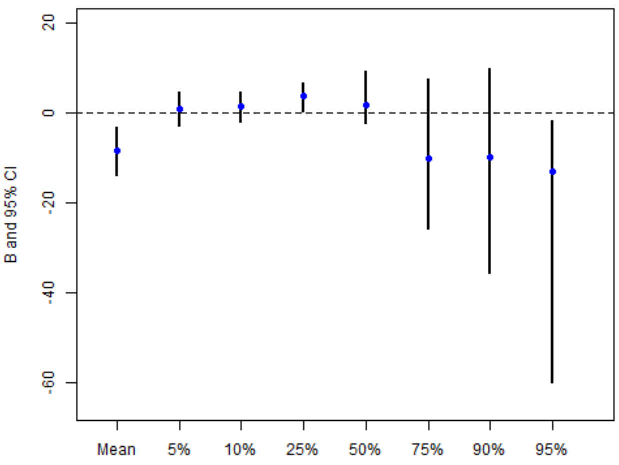

f) aMED

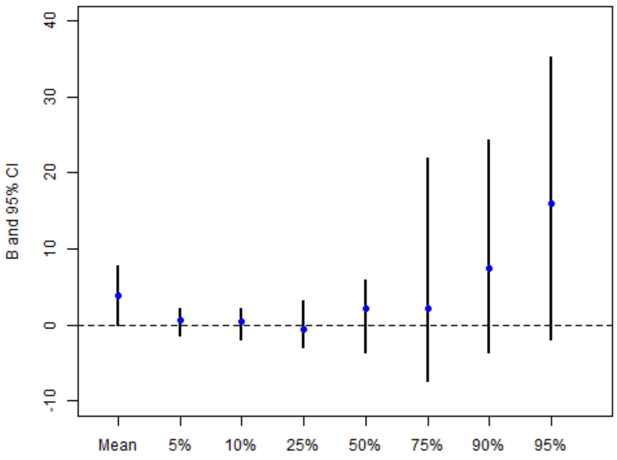

Figure S4. ß estimates[1] from fully adjusted quantile regression plots demonstrating association between dietary pattern scores during pregnancy and child SRS raw scores in NHSII.[2] *Abbreviations: EDIP: Empirical Dietary Inflammatory Pattern, AHEI: Alternative Healthy Eating Index, AHEI-P: Alternative Healthy Eating Index Modified for Pregnancy, aMED: Alternative Mediterranean Diet Score. [1]Estimates for quartile 4 compared to quartile 1. [2]Blue dots indicate the ß from adjusted models including adjustment for maternal age (continuous), child sex (male, female), maternal pre-pregnancy BMI (continuous), household income (0–50k, 50k–100k, 100k+), prenatal vitamin use in first month (yes, no), and total energy intake (continuous). Black lines indicate 95% CIs. †Mutually adjusted for each other

Table S1. Basic characteristics of the NHSII subgroups pregnancy (*n* = 347) and lactation (*n* = 380).

	Pregnancy subgroup	Lactation subgroup
	***n* (%)**	
Child sex		
Male	207 (59.7)	216 (56.8)
Female	140 (40.4)	164 (43.2)
Maternal ethnicity		
Hispanic/Latino	11 (3.2)	3 (0.8)
Not Hispanic/Latino	336 (96.8)	377 (99.2)
Maternal race		
White	337 (97.1)	371 (97.6)
Black/African American	0 (0)	1 (0.3)
Native American or Native Alaskan	–	–
Asian & Pacific Islander	3 (0.9)	6 (1.6)
Multiple/Other Race	5 (1.4)	2 (0.5)
Other/unknown	2 (0.6)	0 (0)
Household income		
$0–40,000 / $0–50,000	11 (3.2)	16 (4.2)
$40,001–100,000 / $50,001–100,000	164 (47.3)	152 (40.0)
$100,001+	122 (35.2)	127 (33.4)
Missing	50 (14.4)	85 (22.4)
Prenatal smoking		
Active	30 (8.7)	21 (5.5)
Passive	–	–
Not active	317 (91.4)	359 (94.5)
Missing	0 (0)	0 (0)
Birthweight		
Low	2 (0.6)	5 (1.3)
Normal	217 (62.5)	219 (57.6)
Missing	128 (36.9)	156 (41.1)
Ever breastfeed		
Yes	311 (89.6)	380 (100)
No	35 (10.1)	0 (0)
Missing	1 (0.3)	0 (0)
Prenatal vitamin use		
Yes	286 (82.4)	246 (64.7)
No	60 (17.3)	134 (35.3)
Missing	1 (0.3)	0 (0)

Subgroups refer to timing of maternal dietary information.

Table S2. Basic characteristics of the study population by high and low quartiles of AHEI-2010 across the two cohorts

	EARLI		NHSII	
	Q1	Q4	Q1	Q4
	n = 40	*n* = 36	*n* = 199	*n* = 175
	n (%)			
Child sex				
Male	25 (62.5)	20 (55.6)	109 (54.8)	109 (62.3)
Female	15 (37.5)	16 (44.4)	90 (45.2)	66 (37.7)
Maternal ethnicity				
Hispanic/Latino	7 (17.5)	11 (30.6)	4 (2.0)	3 (1.7)
Not Hispanic/Latino	33 (82.5)	25 (69.4)	195 (98.0)	172 (92.3)
Maternal race				
White	32 (80)	20 (55.6)	195 (98.0)	172 (98.3)
Black/African American	1 (2.5)	1 (2.8)	1 (0.5)	0 (0)
Native American or Native Alaskan	1 (2.5)	1 (2.8)	0 (0)	0 (0)
Asian & Pacific Islander	3 (7.5)	6 (16.7)	1 (0.5)	1 (0.6)
Multiple/Other Race	2 (5)	5 (13.9)	1 (0.5)	2 (1.1)
Other/unknown	1 (2.5)	3 (8.3)	1 (0.5)	0 (0)
Household income				
\$0–40,000 / \$0–50,000	11 (27.5)	9 (25.0)	8 (4.0)	3 (1.7)
\$40,001–100,000 / \$50,001–100,000	16 (40)	13 (36.1)	100 (50.3)	62 (35.4)
\$100,001+	13 (32.5)	14 (38.9)	54 (27.1)	71 (40.6)
Missing	0 (0)	0 (0)	37 (18.6)	39 (22.3)
Prenatal smoking				
Active	3 (7.5)	1 (2.8)	17 (8.5)	11 (6.3)
Passive	1 (2.5)	0 (0)	–	–
Not active	29 (72.5)	28 (77.8)	182 (91.5)	164 (93.7)
Missing	7 (17.5)	7 (19.4)	0 (0)	0 (0)
Birthweight				
Low	2 (5)	2 (5.6)	0 (0)	3 (1.7)
Normal	38 (95)	34 (94.4)	128 (64.3)	100 (57.1)
Missing	0 (0)	0 (0)	71 (35.7)	72 (41.1)
Ever breastfeed				
Yes	20 (50)	29 (80.6)	186 (93.5)	172 (98.3)
No	13 (32.5)	6 (16.7)	13 (6.5)	3 (1.7)
Missing	7 (17.5)	1 (2.8)	0 (0)	0 (0)
Prenatal vitamin use				
Yes	36 (90.0)	34 (94.4)	142 (71.4)	129 (73.7)
No	4 (10.0)	2 (5.6)	57 (28.6)	46 (26.3)
Missing	0 (0)	0 (0)	0 (0)	0 (0)
Prenatal vitamin use (first month)				
Yes	20 (50.0)	21 (58.3)	–	–
No	20 (50.0)	15 (41.7)	–	–
Missing	0 (0)	0 (0)	–	–
ASD diagnosis				
Yes	12 (30)	8 (22.2)	27 (13.6)	27 (15.4)
No	28 (70)	28 (77.8)	172 (86.4)	148 (84.6)
Missing	0 (0)	0 (0)	0 (0)	0 (0)
	Mean (STD)			
Maternal age, years	33.4 (4.2)	34.6 (4.6)	33.4 (4.2)	35.4 (4.1)
Birthweight (lb)	7.8 (1.3)	7.6 (1.1)	-	-
Parity	1.6 (0.8)	1.7 (0.9)	1.4 (1.3)	1.2 (1.1)
Pre-Pregnancy BMI, kg/m^2	31.9 (8.7)	25.1 (5.4)	23.6 (4.6)	23.0 (3.6)
Physical activity, METs/week	246.3 (371.0)	437.2 (594.8)	14.2 (23.4)	26.5 (26.4)
Total caloric intake, kcal	1683.1 (831.4)	2050.4 (743.4)	2064.4 (538.5)	1810.3 (574.9)

Total SRS raw score	42.7 (27.6)	34.5 (29.5)	26.4 (33.9)	28.9 (32.4)

Abbreviations: EARLI: Early Autism Risk Longitudinal Investigation, NHSII: Nurses' Health Study II, SRS: Social Responsiveness Scale, ASD: Autism Spectrum Disorder, BMI: Body Mass Index, METs: Metabolic Equivalents.

Table S3: Distribution of dietary pattern scores in quartiles across the two cohorts.

	EARLI				NHSII			
	**Q1**	**Q2**	**Q3**	**Q4**	**Q1**	**Q2**	**Q3**	**Q4**
EDIP	−1.48 – −0.06	−0.06 – 0.09	0.10 – 0.34	0.37 – 1.17	−0.80 – −0.03	−0.02 – 0.13	0.13 – 0.30	0.31 – 1.32
AHEI-2010	23.09 – 36.28	36.49 – 42.86	43.08 – 49.85	50.05 – 68.82	15.75 – 35.33	35.46 – 41.72	41.87 – 48.90	49.01 – 75.05
AHEI-P	24.05 – 40.22	40.42 – 52.17	52.29 – 62.80	63.51 – 83.38	20.75 – 41.33	41.40 – 49.76	49.86 – 58.63	58.69 – 84.18
Western	0.44 – 1.35	1.38 – 2.17	2.21 – 2.86	2.87 – 9.11	−2.03 – −0.61	−0.61 – −0.13	−0.13 – 0.47	0.48 – 5.09
Prudent	0.53 – 1.58	1.60 – 2.68	2.70 – 4.19	4.22 – 12.96	−1.88 – −0.58	−0.58 – −0.11	−0.09 - 0.48	0.49 – 6.32
aMED	0 – 1	2 – 3	4 – 4	5 – 8	0 – 2	3 – 4	5 – 5	6 – 8

Abbreviations: EDIP: Empirical Dietary Inflammatory Pattern, AHEI: Alternative Healthy Eating Index, AHEI-P: Alternative Healthy Eating Index. Modified for Pregnancy, aMED: Alternative Mediterranean Diet Score. See text for further descriptions of dietary patterns.

Table S4: Distribution of total energy intake (mean, SD) by dietary patterns in EARLI and NHSII

	Q1	Q2	Q3	Q4
	**EARLI**			
EDIP	1847.28 (638.1)	1489.38 (625.8)	1660.70 (843.3)	2286.34 (861.5)
AHEI-2010	1683.13 (831.4)	1634.18 (692.5)	1985.31 (875.5)	2050.38 (743.4)
AHEI-P	1132.70 (345.8)	1641.69 (525.5)	2178.30 (695.9)	2447.63 (841.7)
Western	1144.82 (408.5)	1401.65 (282.2)	1861.28 (474.3)	2798.97 (694.2)
Prudent	1335.55 (559.6)	1619.23 (649.3)	1986.31 (710.9)	2390.97 (850.7)
aMED	1447.29 (518.7)	1532.17 (685.3)	1909.23 (547.0)	2562.00 (858.9)
	**NHSII**			
EDIP	1910.05 (545.90)	1831.23 (465.30)	1874.11 (543.64)	2171.43 (580.56)
AHEI-2010	2064.38 (538.48)	2000.52 (544.72)	1892.62 (506.70)	1810.26 (574.88)
AHEI-P	1563.26 (376.74)	1869.44 (472.19)	2014.53 (482.77)	2338.93 (535.84)
Western	1507.64 (393.87)	1735.22 (367.11)	1994.19 (420.40)	2475.89 (465.04)
Prudent	1672.60 (451.74)	1846.47 (507.53)	2049.85 (511.86)	2252.10 (548.32)
aMED	1648.90 (429.78)	1894.41 (537.94)	2135.95 (531.42)	2165.23 (537.15)

Abbreviations: EDIP: Empirical Dietary Inflammatory Pattern, AHEI: Alternative Healthy Eating Index, AHEI-P: Alternative Healthy Eating Index Modified for Pregnancy, aMED: Alternative Mediterranean Diet Score. Table shows the mean and standard deviation of total energy intake (total calories) for each quartile of each dietary pattern in the two cohorts.

Table S5: Association between maternal dietary patterns during pregnancy and child SRS raw scores in EARLI, adjusting for additional potential confounders (*n* = 131)

	n	**Fully Adjusted Model + parity**	**Fully Adjusted Model + physical activity**	**Fully Adjusted Model + maternal race/ethnicity**	**Fully Adjusted Model + smoking status**
EDIP					
Q1	31	0 (reference)	0 (reference)	0 (reference)	0 (reference)
Q2	35	−8.38 (−13.54, 4.53)	−7.80 (−12.34, 4.81)	−7.58 (−14.93, 4.60)	−7.70 (−13.49, 3.00)
Q3	29	−7.93 (−18.04, 2.57)	−6.98 (−16.96, 2.67)	−7.61 (−15.28, 4.55)	−6.49 (−15.08, 2.08)
Q4	36	−6.85 (−10.21, 7.09)	−5.30 (−15.36, 5.88)	−6.58 (−11.37, 6.63)	−4.55 (−8.81, 6.16)
AHEI-2010					
Q1	35	0 (reference)	0 (reference)	0 (reference)	0 (reference)
Q2	40	−6.92 (−13.62, 2.52)	−6.11 (−14.38, 6.52)	−6.70 (−14.13, 6.13)	−4.32 (−12.42, 4.85)
Q3	24	−6.22 (−13.26, 6.85)	−4.65 (−11.02, 6.99)	−5.64 (−11.54, 5.92)	−4.08 (−12.01, 10.38)
Q4	32	−6.44 (−20.02, 10.24)	−5.18 (−21.84, 9.51)	−6.82 (−23.09, 10.61)	−4.26 (−20.17, 11.16)
AHEI-P					
Q1	34	0 (reference)	0 (reference)	0 (reference)	0 (reference)
Q2	33	3.02 (−10.66, 15.77)	4.17 (−11.29, 12.56)	−0.02 (−10.89, 14.99)	5.02 (−11.41, 14.83)
Q3	34	0.58 (−17.78, 18.23)	3.00 (−18.94, 14.46)	−3.30 (−15.28, 17.53)	2.84 (−13.75, 17.35)
Q4	30	−8.02 (−26.65, 8.10)	−3.01 (−24.37, 9.18)	−11.27 (−25.03, 11.59)	−4.11 (−23.65, 11.25)
Western†					
Q1	30	0 (reference)	0 (reference)	0 (reference)	0 (reference)
Q2	32	14.77 (2.89, 23.35)	12.05 (5.08, 23.16)	14.38 (−0.18, 25.35)	11.36 (2.48, 19.94)
Q3	33	8.50 (−4.10, 15.15)	2.24 (−7.31, 15.16)	8.67 (−12.41, 16.55)	4.41 (−5.25, 15.24)
Q4	36	8.52 (−18.70, 25.46)	9.92 (−17.80, 22.86)	11.91 (−19.99, 24.39)	7.52 (−15.02, 24.33)
Prudent†					
Q1	33	0 (reference)	0 (reference)	0 (reference)	0 (reference)
Q2	35	−9.26 (−15.36, 8.86)	−5.49 (−13.14, 7.39)	−7.66 (−17.87, 7.80)	−5.26 (−12.62, 8.53)
Q3	31	−3.56 (−12.23, 12.30)	−6.23 (−12.34, 13.67)	−5.47 (−12.52, 14.57)	−1.39 (−9.66, 15.78)
Q4	32	−14.80 (−23.07, 1.23)	−10.78 (−22.85, 2.61)	−13.70 (−25.13, 2.67)	−11.38 (−20.10, 2.62)
aMED					
Q1	20	0 (reference)	0 (reference)	0 (reference)	0 (reference)
Q2	55	−9.11 (−13.66, 6.74)	−7.28 (−12.10, 2.73)	−8.80 (−14.12, 4.73)	−8.47 (−12.36, 2.10)
Q3	27	0.04 (−8.07, 10.00)	1.24 (−8.20, 9.17)	−0.10 (−9.41, 9.98)	1.71 (−8.43, 13.17)
Q4	29	−12.64 (−23.51, 5.27)	−11.86 (−23.14, 5.50)	−10.86 (−27.38, 4.53)	−15.38 (−21.96, 7.70)

Abbreviations: EDIP: Empirical Dietary Inflammatory Pattern, AHEI: Alternative Healthy Eating Index, AHEI-P: Alternative Healthy Eating Index Modified for Pregnancy, aMED: Alternative Mediterranean Diet Score. Modeled using quantile regression fixed at 50th percentile Fully Adjusted: adjusted for maternal age (continuous), child sex (male, female), maternal pre-pregnancy BMI (continuous), household income (0–50k, 50k–100k, 100k+), prenatal vitamin use in first month (yes, no), and total energy intake (continuous). †Mutually adjusted for each other

Table S6: Association between maternal dietary patterns and child SRS raw scores in NHSII, adjusted for additional potential confounders (*n* = 727)

	Fully adjusted + year of birth (ß, 95% CI)	Fully adjusted + parity (ß, 95% CI)	Fully adjusted + physical activity (ß, 95% CI)	Fully adjusted + maternal race/ethnicity (ß, 95% CI)	Fully adjusted + smoking status (ß, 95% CI)
EDIP					
Q1	0 (reference)	0 (reference)	0 (reference)	0 (reference)	0 (reference)
Q2	−4.01 (−7.95, −0.31)	−4.13 (−6.50, −0.38)	−4.20 (−6.68, −0.66)	−4.00 (−6.73, −0.79)	−4.00 (−6.90, −0.42)
Q3	−2.42 (−5.09, 0.13)	−1.98 (−4.40, 0.75)	−1.79 (−4.38, 1.46)	−2.16 (−4.48, 1.16)	−1.39 (−4.45, 1.19)
Q4	−0.33 (−3.19, 3.55)	0.61 (−2.32, 3.18)	1.06 (−2.31, 3.33)	0.49 (−3.34, 2.90)	1.16 (−2.53, 3.45)
AHEI-2010					
Q1	0 (reference)	0 (reference)	0 (reference)	0 (reference)	0 (reference)
Q2	−0.08 (−2.03, 2.06)	−0.32 (−3.03, 4.27)	0.33 (−2.86, 4.21)	−0.72 (−2.76, 3.00)	0.36 (−2.71, 4.19)
Q3	0.45 (−2.61, 3.03)	−0.09 (−3.40, 3.67)	0.46 (−3.53, 3.56)	−0.25 (−3.43, 3.22)	0.58 (−3.49, 3.46)
Q4	2.61 (−0.46, 6.69)	1.46 (−1.56, 6.73)	2.50 (−2.40, 6.87)	1.77 (−1.89, 7.05)	2.62 (−1.94, 7.05)
AHEI-P					
Q1	0 (reference)	0 (reference)	0 (reference)	0 (reference)	0 (reference)
Q2	−1.46 (−5.91, 1.60)	−1.15 (−5.40, 2.13)	−1.39 (−6.59, 1.63)	−1.60 (−5.51, 1.63)	−1.23 (−5.23, 1.79)
Q3	−1.31 (−5.04, 1.16)	−1.37 (−4.73, 2.29)	−1.68 (−5.27, 1.12)	−1.70 (−4.99, 1.18)	−1.52 (−4.47, 1.26)
Q4	1.06 (−2.56, 4.50)	1.54 (−3.02, 5.71)	1.25 (−4.29, 4.66)	1.33 (−3.61, 4.89)	1.83 (−3.07, 4.42)
Western†					
Q1	0 (reference)	0 (reference)	0 (reference)	0 (reference)	0 (reference)
Q2	1.96 (−1.90, 5.82)	2.48 (0.05, 7.57)	2.75 (−0.56, 7.62)	2.19 (−0.78, 7.42)	3.11 (−0.61, 7.60)
Q3	1.64 (−2.78, 6.07)	1.93 (−1.60, 6.43)	1.82 (−2.49, 7.14)	0.72 (−2.66, 7.07)	1.81 (−2.39, 7.12)
Q4	2.86 (−2.60, 8.31)	2.36 (−1.54, 9.71)	1.95 (−2.58, 8.80)	1.19 (−1.96, 9.07)	1.97 (−2.17, 9.72)
Prudent†					
Q1	0 (reference)	0 (reference)	0 (reference)	0 (reference)	0 (reference)
Q2	−1.86 (−5.44, 1.72)	−1.25 (−5.05, 2.62)	−1.71 (−5.83, 2.51)	−1.49 (−4.83, 2.09)	−1.09(−5.11, 2.66)
Q3	0.00 (−3.32, 3.32)	0.17 (−3.11, 4.37)	−0.30 (−3.35, 4.29)	−0.21(−3.63, 3.94)	0.14 (−4.08, 3.55)
Q4	2.31 (−1.99, 6.61)	2.85 (−1.31, 8.11)	1.87 (−1.66, 7.62)	2.28 (−1.88, 8.07)	2.64 (−1.80, 7.87)
aMED					
Q1	0 (reference)	0 (reference)	0 (reference)	0 (reference)	0 (reference)
Q2	0.39 (−3.05, 2.66)	−0.04 (−1.69, 3.86)	0.43 (−1.86, 3.79)	0.55 (−1.62, 3.87)	0.29 (−2.14, 4.01)
Q3	−0.64 (−5.45, 2.53)	−0.70 (−4.64, 3.84)	−0.55 (−4.92, 3.96)	−0.27 (−4.07, 4.43)	−0.45 (−5.08, 4.48)
Q4	1.01 (−2.02, 5.26)	1.35 (−2.90, 5.41)	2.11 (−3.77, 5.52)	2.45 (−3.60, 5.25)	2.19 (−3.69, 6.23)

Abbreviations: EDIP: Empirical Dietary Inflammatory Pattern, AHEI: Alternative Healthy Eating Index, AHEI-P: Alternative Healthy Eating Index Modified for Pregnancy, aMED: Alternative Mediterranean Diet Score. Modeled using quantile regression fixed at 50th percentile. Fully Adjusted Model: Adjusted for maternal age (continuous), child sex (male, female), maternal pre-pregnancy BMI (continuous), household income (0–40k, 40k–100k, 100k+), prenatal vitamin use (yes, no), and total energy intake (continuous).
†Mutually adjusted for each other

Table S7: Association between maternal dietary patterns during pregnancy and child autism spectrum disorders in EARLI (*n* = 146)

	Cases/*n*	Crude (OR, 95% CI)	Adjusted (OR, 95% CI)	Fully Adjusted (OR, 95% CI)
EDIP				
Q1 + Q2	17/76	1 (reference)	1 (reference)	1 (reference)
Q3 + Q4	13/70	0.79 (0.35, 1.78)	0.83 (0.34, 2.01)	0.93 (0.37, 2.30)
AHEI-2010				
Q1 + Q2	16/76	1 (reference)	1 (reference)	1 (reference)
Q3 + Q4	14/70	0.94 (0.42, 2.10)	1.29 (0.52, 3.17)	1.52 (0.59, 3.87)
AHEI-P				
Q1 + Q2	18/75	1 (reference)	1 (reference)	1 (reference)
Q3 + Q4	12/71	0.64 (0.28, 1.46)	0.71 (0.29, 1.74)	0.97 (0.33, 2.86)
Western†				
Q1 + Q2	18/73	1 (reference)	1 (reference)	1 (reference)
Q3 + Q4	12/73	0.60 (0.27, 1.36)	0.58 (0.24, 1.40)	0.71 (0.22, 2.31)
Prudent†				
Q1 + Q2	17/75	1 (reference)	1 (reference)	1 (reference)
Q3 + Q4	13/71	0.76 (0.34, 1.72)	1.05 (0.41, 2.72)	1.19 (0.41, 3.44)
aMED				
Q1 + Q2	17/83	1 (reference)	1 (reference)	1 (reference)
Q3 + Q4	13/63	1.01 (0.45, 2.27)	1.34 (0.54, 3.31)	2.03 (0.72, 5.77)

Abbreviations: EDIP: Empirical Dietary Inflammatory Pattern, AHEI: Alternative Healthy Eating Index, AHEI-P: Alternative Healthy Eating Index Modified for Pregnancy, aMED: Alternative Mediterranean Diet Score. Modeled using logistic regression. Adjusted: Adjusted for maternal age (continuous), child sex (male, female), maternal pre-pregnancy BMI (continuous), household income (0–50k, 50k−100k, 100k+), prenatal vitamin use in first month (yes, no). Fully Adjusted: Adjusted Model, additionally adjusted for total energy intake (continuous). †Mutually adjusted for each other

Table S8: Association between maternal dietary patterns during pregnancy and child SRS T scores in EARLI (*n* = 131) and NHSII (*n* = 727)

	EARLI		NHSII	
	n	**Fully Adjusted (β, 95% CI)**	***n***	**Fully Adjusted (β, 95% CI)**
EDIP				
Q1	31	0 (reference)	167	0 (reference)
Q2	35	−2.76 (−6.34, 0.23)	200	−1.22 (−2.38, 0.60)
Q3	29	−2.76 (−5.76, 0.74)	175	−0.91 (−2.13, 1.06)
Q4	36	−1.72 (−5.72, 1.58)	185	0.65 (−1.03, 2.67)
AHEI-2010				
Q1	35	0 (reference)	199	0 (reference)
Q2	40	−4.23 (−5.71, 0.96)	178	0.11 (−0.97, 2.70)
Q3	24	−3.16 (−4.80, 3.53)	175	0.33 (−1.37, 1.80)
Q4	32	−2.54 (−7.10, 4.09)	175	1.25 (−1.13, 3.29)
AHEI-P				
Q1	34	0 (reference)	193	0 (reference)
Q2	33	0.40 (−5.40, 4.66)	169	−0.58(−2.66, 0.98)
Q3	34	0.16 (−6.58, 4.93)	174	−0.41(−2.68, 0.92)
Q4	30	−2.07 (−8.95, 5.46)	191	0.31(−2.46, 2.77)
Western†				
Q1	30	0 (reference)	168	0 (reference)
Q2	32	3.94 (1.25, 9.40)	178	1.62 (−0.81, 3.68)
Q3	33	0.59 (−3.32, 5.42)	188	1.34 (−1.87, 3.27)
Q4	36	1.15 (−4.11, 7.04)	193	1.44 (−1.30, 4.77)
Prudent†				
Q1	33	0 (reference)	197	0 (reference)
Q2	35	−3.66 (−6.43, 0.62)	182	−0.99 (−2.31, 0.96)
Q3	31	−3.11 (−5.73, 3.64)	170	0.07 (−2.31, 0.96)
Q4	32	−6.22 (−9.65, 1.57)	178	1.45 (−1.57, 2.52)
aMED				
Q1	20	0 (reference)	170	0 (reference)
Q2	55	−3.61 (−6.50, 2.57)	248	0.34 (−1.08, 1.73)
Q3	27	−0.34 (−5.11, 4.38)	146	0.54 (−1.96, 3.22)
Q4	29	−3.74 (−9.09, 2.55)	163	0.78 (−1.82, 2.74)

Abbreviations: EDIP: Empirical Dietary Inflammatory Pattern, AHEI: Alternative Healthy Eating Index, AHEI-P: Alternative Healthy Eating Index Modified for Pregnancy, aMED: Alternative Mediterranean Diet Score. Modeled using quantile regression fixed at 50th percentile. Fully Adjusted: adjusted for maternal age (continuous), child sex (male, female), maternal pre-pregnancy BMI (continuous), household income (EARLI: 0–50k, 50k–100k, 100k+, NHSII: 0–40k, 40k–100k, 100k+), prenatal vitamin use (yes, no) (EARLI: in first month of pregnancy, NHSII: during pregnancy), and total energy intake (continuous). †Mutually adjusted for each other

Table S9: Full quantile regression results for the association between maternal dietary patterns and child SRS scores in EARLI (*n* = 131)

		Fully Adjusted (β, 95% CI)			
	n	**25th percentile**	**50th percentile**	**75th percentile**	**90th percentile**
EDIP					
Q1	31	0 (reference)	0 (reference)	0 (reference)	0 (reference)
Q2	35	−5.74 (−13.85, 4.51)	−8.29 (−14.49, 3.02)	−4.79 (−28.96, 24.38)	−4.97 (−46.87, 48.07)
Q3	29	−6.58 (−14.32, 7.53)	−7.72 (−15.58, 3.80)	−8.89 (−31.29, 18.22)	−17.53 (−49.63, 12.04)
Q4	36	−4.61 (−13.37, 7.75)	−6.70 (−10.71, 6.55)	−17.41 (−34.98, 0.84)	−28.88 (−66.00, 2.40)
AHEI-2010					
Q1	35	0 (reference)	0 (reference)	0 (reference)	0 (reference)
Q2	40	−4.76 (−11.84, 4.85)	−6.30 (−14.38, 3.91)	−4.11 (−25.87, 7.87)	−12.65 (−56.96, 4.34)
Q3	24	−2.86 (−8.90, 9.82)	−5.86 (−11.45, 5.10)	−4.37 (−26.09, 2.71)	−6.80 (−48.52, 49.71)
Q4	32	−8.61 (−18.97, 2.90)	−6.03 (−23.11, 11.96)	−0.23 (−32.38, 16.71)	1.00 (−41.94, 71.01)
AHEI-P					
Q1	34	0 (reference)	0 (reference)	0 (reference)	0 (reference)
Q2	33	−1.10 (−9.95, 9.18)	3.70 (−10.91, 14.04)	−0.37 (−29.15, 25.01)	22.99 (−30.93, 55.43)
Q3	34	−4.62 (−10.50, 8.12)	0.02 (−16.34, 17.43)	−14.34 (−33.59, 3.96)	−11.21 (−43.63, 4.38)
Q4	30	−7.62 (−19.17, 4.71)	−7.58 (−25.64, 9.14)	−16.03 (−43.06, 8.30)	−19.37 (−40.86, 7.88)
Western†					
Q1	30	0 (reference)	0 (reference)	0 (reference)	0 (reference)
Q2	32	14.10 (1.20, 21.55)	11.71 (2.16, 22.71)	7.63 (−14.49, 39.95)	7.35 (−46.57, 59.53)
Q3	33	4.28 (−4.56, 15.13)	5.29 (−6.85, 16.77)	−6.27 (−24.94, 14.95)	−24.04 (−64.14, 4.90)
Q4	36	4.10 (−15.90, 14.85)	8.01 (−19.90, 22.14)	−2.23 (−24.76, 16.87)	−42.83 (−47.84, 58.90)
Prudent†					
Q1	33	0 (reference)	0 (reference)	0 (reference)	0 (reference)
Q2	35	−1.32 (−11.42, 11.49)	−7.69 (−13.31, 7.30)	−13.28 (−47.13, −2.18)	−23.60 (−41.64, 24.96)
Q3	31	2.75 (−12.20, 11.02)	−6.04 (−12.14, 14.62)	−8.78 (−40.93, 5.90)	−22.58 (−55.29, −1.86)
Q4	32	−7.18 (−15.11, 2.41)	−14.17 (−22.76, 2.11)	−15.90 (−49.37, 2.17)	−22.18 (−47.32, 4.64)
aMED					
Q1	20	0 (reference)	0 (reference)	0 (reference)	0 (reference)
Q2	55	−2.23 (−15.57, 9.73)	−9.02 (−12.88, 5.85)	−1.48 (−34.16, 10.37)	*Did not converge*
Q3	27	−1.11 (−24.82, 16.26)	0.24 (−8.50, 9.99)	0.05 (−34.30, 12.36)	62.55 (−41.48, 87.05)
Q4	29	−7.19 (−20.93, 6.26)	−12.32 (−23.33, 4.05)	−2.41 (−50.54, 14.17)	10.67 (−58.03, 47.89)

Abbreviations: EDIP: Empirical Dietary Inflammatory Pattern, AHEI: Alternative Healthy Eating Index, AHEI-P: Alternative Healthy Eating Index Modified for Pregnancy, aMED: Alternative Mediterranean Diet Score. Modeled using quantile regression. Fully Adjusted: adjusted for maternal age (continuous), child sex (male, female), maternal pre-pregnancy BMI (continuous), household income (0–50k, 50k−100k, 100k+), prenatal vitamin use in first month (yes, no), and total energy intake (continuous). †Mutually adjusted for each other

Table S10: Full quantile regression results for the association between maternal dietary patterns and child SRS scores in NHSII (*n* = 727)

		Fully Adjusted (β, 95% CI)			
	n	**25th percentile**	**50th percentile**	**75th percentile**	**90th percentile**
EDIP					
Q1	167	0 (reference)	0 (reference)	0 (reference)	0 (reference)
Q2	200	−2.14 (−4.13, 0.57)	−3.94 (−6.71, −0.58)	−0.73 (−5.30, 6.06)	2.12 (−14.14, 15.68)
Q3	175	−1.53 (−3.50, 1.91)	−1.92 (−4.44, 1.17)	−1.54 (−8.18, 7.47)	1.99 (−15.61, 13.68)
Q4	185	−0.28 (−2.31, 3.52)	0.96 (−2.74, 3.17)	10.43 (−0.07, 21.48)	14.90 (−4.25, 24.73)
AHEI-2010					
Q1	199	0 (reference)	0 (reference)	0 (reference)	0 (reference)
Q2	178	0.50 (−1.27, 2.80)	0.36 (−2.78, 4.22)	0.35 (−6.40, 11.07)	7.47 (−7.62, 27.70)
Q3	175	−0.09 (−2.03, 2.54)	0.13 (−3.61, 3.46)	3.46 (−6.71, 12.25)	7.87 (−8.85, 20.86)
Q4	175	0.50 (−1.29, 2.17)	2.62 (−2.03, 6.93)	1.84 (−5.17, 13.98)	5.16 (−7.10, 20.81)
AHEI-P					
Q1	193	0 (reference)	0 (reference)	0 (reference)	0 (reference)
Q2	169	−0.66 (−3.27, 1.60)	−1.31 (−5.51, 1.72)	−9.04 (−15.85, −0.57)	−1.31 (−9.94, 10.98)
Q3	174	−1.48 (−3.18, 0.55)	−1.61 (−4.53, 1.06)	−7.54 (−16.37, 6.82)	−0.10 (−8.64, 13.02)
Q4	191	−1.41 (−3.95, 1.53)	1.51 (−3.32, 4.44)	−5.52 (−14.03, 4.34)	14.55 (−7.59, 32.27)
Western†					
Q1	168	0 (reference)	0 (reference)	0 (reference)	0 (reference)
Q2	178	2.75 (−0.01, 4.75)	2.71 (−0.31, 7.73)	−4.64 (−16.42, 7.67)	−8.11 (−25.14, 6.86)
Q3	188	1.21 (−1.55, 3.36)	1.68 (−2.46, 6.78)	−6.55 (−19.32, 8.13)	−13.59 (−34.88, −0.82)
Q4	193	3.86 (0.29, 6.71)	1.83 (−2.35, 9.38)	−9.99 (−25.65, 7.70)	−9.80 (−35.50, 9.87)
Prudent†					
Q1	197	0 (reference)	0 (reference)	0 (reference)	0 (reference)
Q2	182	−0.34 (−2.08, 1.47)	−1.09 (−4.81, 2.89)	−10.56 (−16.94, −0.15)	−10.01 (−23.20, 2.31)
Q3	170	0.27 (−2.21, 1.67)	0.23 (−4.04, 3.93)	−8.29 (−15.82, 12.46)	−2.99 (−24.73, 17.22)
Q4	178	0.59 (−2.07, 3.06)	2.73 (−1.92, 8.16)	−8.70 (−19.78, 8.74)	−1.81 (−27.07, 21.09)
aMED					
Q1	170	0 (reference)	0 (reference)	0 (reference)	0 (reference)
Q2	248	−0.22 (−1.55, 2.78)	0.27 (−1.76, 3.87)	1.97 (−8.52, 8.16)	−1.47 (−9.00, 14.91)
Q3	146	−1.20 (−3.46, 2.09)	−0.49 (−4.75, 4.51)	−2.41 (−10.99, 6.09)	−5.71 (−14.70, 18.74)
Q4	163	−0.61 (−2.93, 3.14)	2.16 (−3.70, 5.98)	2.17 (−7.46, 21.93)	7.40 (−3.65, 24.39)

Abbreviations: EDIP: Empirical Dietary Inflammatory Pattern, AHEI: Alternative Healthy Eating Index, AHEI-P: Alternative Healthy Eating Index Modified for Pregnancy, aMED: Alternative Mediterranean Diet Score. Modeled using quantile regression. Fully Adjusted Model: Adjusted for maternal age (continuous), child sex (male, female), maternal pre-pregnancy BMI (continuous), household income (0–40k, 40k−100k, 100k+), prenatal vitamin use (yes, no), and total energy intake (continuous). †Mutually adjusted for each other

Table S11: Associations between maternal dietary patterns and child SRS scores in the NHSII and EARLI cohorts, stratified by child's sex

	EARLI				NHSII			
	n	Male Fully Adjusted (ß, 95% CI)	*n*	Female Fully Adjusted (ß, 95% CI)	*n*	Male Fully Adjusted (ß, 95% CI)	*n*	Female Fully Adjusted (ß, 95% CI)
EDIP								
Q1	19	0 (reference)	12	0 (reference)	101	0 (reference)	66	0 (reference)
Q2	19	−19.07 (−25.91, 14.32)	16	−0.94 (−16.07, 19.00)	109	−5.39 (−15.09, −2.84)	91	−0.34 (−4.57, 4.63)
Q3	12	−16.25 (−28.13, −1.20)	17	−1.98 (−17.28, 10.57)	107	−1.57 (−12.10, 6.61)	68	−1.88 (−4.09, 2.50)
Q4	20	−13.74 (−25.74, 14.88)	16	−6.10 (−24.29, 6.65)	106	2.52 (−9.48, 6.48)	79	−1.33 (−3.41, 4.06)
AHEI-2010								
Q1	21	0 (reference)	14	0 (reference)	109	0 (reference)	90	0 (reference)
Q2	22	−13.59 (−41.78, 1.79)	18	−3.87 (−11.35, 11.08)	102	2.40 (−4.74, 10.22)	76	−0.91 (−3.49, 2.28)
Q3	9	−3.26 (−30.86, 12.95)	15	−7.04 (−13.85, 8.46)	103	−2.43 (−6.20, 7.20)	72	2.14 (−1.57, 4.67)
Q4	18	−11.00 (−38.50, 3.89)	14	−3.99 (−22.21, 16.90)	109	4.09 (−4.03, 14.06)	66	1.55 (−2.94, 5.21)
AHEI-P								
Q1	19	0 (reference)	15	0 (reference)	106	0 (reference)	87	0 (reference)
Q2	15	−5.05 (−35.78, 21.76)	18	6.95 (−11.55, 19.21)	92	−0.32 (−8.33, 5.09)	77	−1.43 (−6.04, 1.52)
Q3	17	−2.69 (−32.26, 23.33)	17	−5.09 (−22.01, 20.46)	108	0.44 (−8.32, 8.23)	66	−3.01 (−7.01, 0.10)
Q4	19	−20.56 (−49.21, 15.69)	11	1.21 (−27.90, 17.47)	117	3.60 (−7.35, 11.44)	74	1.37 (−4.75, 4.60)
Western†								
Q1	14	0 (reference)	16	0 (reference)	96	0 (reference)	72	0 (reference)
Q2	19	10.35 (4.11, 27.94)	13	0.92 (−12.97, 21.73)	107	6.64 (−5.32, 11.22)	71	3.09 (−2.44, 7.06)
Q3	16	10.52 (−19.03, 32.56)	17	−8.37 (−23.76, 15.38)	105	3.08 (−10.98, 8.73)	83	3.28 (−4.09, 8.04)
Q4	21	6.93 (−17.94, 28.65)	15	−8.65 (−27.12, 25.27)	115	4.13 (−8.53, 13.34)	78	3.38 (−2.38, 10.12)
Prudent†								
Q1	20	0 (reference)	13	0 (reference)	113	0 (reference)	84	0 (reference)
Q2	15	−4.33 (−33.48, 8.60)	20	−7.93 (−18.23, 6.92)	101	−2.36 (−8.90, 4.14)	81	−0.79 (−5.72, 2.05)
Q3	14	3.88 (−23.88, 19.11)	17	−10.51 (−25.11, 13.92)	102	3.84 (−5.48, 9.89)	68	−1.50 (−5.22, 2.66)
Q4	21	−14.34 (−39.32, 5.36)	11	−16.94 (−25.58, −0.52)	107	4.78 (−6.43, 10.22)	71	1.71 (−3.48, 8.29)
aMED								
Q1	14	0 (reference)	6	0 (reference)	95	0 (reference)	75	0 (reference)
Q2	26	−3.57 (−31.27, 19.47)	29	−6.72 (−15.18, 10.61)	134	3.05 (−3.47, 8.74)	114	−0.24 (−2.72, 3.44)
Q3	15	1.53 (−15.69, 25.87)	12	1.79 (−8.41, 22.73)	98	−1.51 (−8.00, 6.29)	48	0.76 (−3.18, 4.71)
Q4	15	−19.13 (−39.55, −9.36)	14	−0.09 (−13.47, 22.99)	96	4.41 (−5.60, 13.12)	67	2.57 (−3.47, 5.27)

Abbreviations: EDIP: Empirical Dietary Inflammatory Pattern, AHEI: Alternative Healthy Eating Index, AHEI-P: Alternative Healthy Eating Index Modified for Pregnancy, aMED: Alternative Mediterranean Diet Score. Modeled using quantile regression fixed at 50th percentile. Fully Adjusted: adjusted for maternal age (continuous), child sex (male, female), maternal pre-pregnancy BMI (continuous), household income (EARLI: 0–50k, 50k–100k, 100k+, NHSII: 0–40k, 40k–100k, 100k+), prenatal vitamin use (yes, no) (EARLI: in first month of pregnancy, NHSII: during pregnancy), and total energy intake (continuous). †Mutually adjusted for each other

Table S12: Association between maternal dietary patterns and child SRS raw scores in NHSII subgroups

	Total NHSII sample Fully Adjusted (*n* = 727)	Pregnancy subgroup Fully Adjusted (*n* = 347)		Lactation subgroup Fully Adjusted (*n* = 380)	
EDIP	**(ß, 95% CI)**	***n***	**(ß, 95% CI)**	***n***	**(ß, 95% CI)**
Q1	0 (reference)	81	0 (reference)	86	0 (reference)
Q2	−3.94 (−6.71, −0.58)	89	−5.64 (−9.01, 2.44)	111	−1.77 (−6.05, 1.20)
Q3	−1.92 (−4.44, 1.17)	88	−5.95 (−10.61, 2.24)	87	0.45 (−3.63, 3.97)
Q4	0.96 (−2.74, 3.17)	89	0.79 (−2.93, 7.71)	96	1.23 (−2.79, 4.99)
AHEI-2010					
Q1	0 (reference)	105	0 (reference)	94	0 (reference)
Q2	0.36 (−2.78, 4.22)	88	4.64 (−1.34, 8.77)	90	−2.72 (−6.08, 2.11)
Q3	0.13 (−3.61, 3.46)	77	1.26 (−5.28, 6.39)	98	−1.29 (−4.62, 4.05)
Q4	2.62 (−2.03, 6.93)	77	1.98 (−3.78, 8.45)	98	0.48 (−2.13, 5.48)
AHEI-P					
Q1	0 (reference)	86	0 (reference)	107	0 (reference)
Q2	−1.31 (−5.51, 1.72)	89	−1.71 (−7.31, 6.61)	80	−4.15 (−7.16, −0.21)
Q3	−1.61 (−4.53, 1.06)	73	−2.77 (−8.54, 6.68)	101	−1.92 (−5.95, 1.58)
Q4	1.51 (−3.32, 4.44)	99	1.96 (−5.03, 8.01)	92	−1.34 (−4.66, 4.36)
Western†					
Q1	0 (reference)	73	0 (reference)	95	0 (reference)
Q2	2.71 (−0.31, 7.73)	80	10.02 (−2.25, 17.52)	98	−1.26 (−4.69, 1.68)
Q3	1.68 (−2.46, 6.78)	94	3.29 (−5.37, 8.92)	94	1.93 (−2.78, 5.44)
Q4	1.83 (−2.35, 9.38)	100	7.67 (−8.13, 13.32)	93	2.07 (−1.86, 6.51)
Prudent†					
Q1	0 (reference)	93	0 (reference)	104	0 (reference)
Q2	−1.09 (−4.81, 2.89)	90	−1.67 (−8.90, 4.28)	92	−1.72 (−6.39, 2.19)
Q3	0.23 (−4.04, 3.93)	79	0.92 (−6.99, 11.23)	91	−1.33 (−4.23, 1.51)
Q4	2.73 (−1.92, 8.16)	85	4.68 (−8.49, 11.21)	93	1.75 (−2.70, 6.17)
AMED					
Q1	0 (reference)	85	0 (reference)	85	0 (reference)
Q2	0.27 (−1.76, 3.87)	113	−0.75 (−6.46, 6.25)	135	1.45 (−2.05, 5.28)
Q3	−0.49 (−4.75, 4.51)	68	−2.71 (−8.34, 6.32)	78	−0.17 (−4.87, 7.53)
Q4	2.16 (−3.70, 5.98)	81	5.47 (−4.53, 14.50)	82	1.05 (−3.00, 3.24)

Abbreviations: EDIP: Empirical Dietary Inflammatory Pattern, AHEI: Alternative Healthy Eating Index, AHEI-P: Alternative Healthy Eating Index Modified for Pregnancy, aMED: Alternative Mediterranean Diet Score. Modeled using quantile regression fixed at 50th percentile. Total NHSII sample is the primary NHSII population shown in main results tables, copied here for ease of comparison. Fully Adjusted: adjusted for maternal age (continuous), child sex (male, female), maternal pre-pregnancy BMI (continuous), household income (0–40k, 40k–100k, 100k+), prenatal vitamin use (yes, no), and total energy intake (continuous). †Mutually adjusted for each other
